# PDCD4 controls the G1/S-phase transition in a telomerase-immortalized epithelial cell line and affects the expression level and translation of multiple mRNAs

**DOI:** 10.1038/s41598-020-59678-w

**Published:** 2020-02-17

**Authors:** Astrid Haas, Benedikt S. Nilges, Sebastian A. Leidel, Karl-Heinz Klempnauer

**Affiliations:** 1 Institute for Biochemistry, Westfälische-Wilhelms-Universität Münster, Wilhelm-Klemm-Str. 2, D-48149 Münster, Germany; 2 Max Planck Research Group for RNA Biology, Max Planck Institute for Molecular Biomedicine, Röntgenstrasse 20, D-48149 Münster, Germany; 3 Present Address: Department for Chemistry and Biochemistry, University of Bern, Freiestrasse 3, CH-3012 Bern, Switzerland

**Keywords:** DNA synthesis, Ribosome, Tumour-suppressor proteins

## Abstract

PDCD4, the protein encoded by the tumor suppressor gene *PDCD4* (*programmed cell death 4*) has been implicated in the control of cellular transcription and translation by modulating the activity of specific transcription factors and suppressing the translation of mRNAs with structured 5′-UTRs. Most studies of human PDCD4 have employed tumor cell lines, possibly resulting in a biased picture of its role in normal cells. Here, we have studied the function of PDCD4 in a telomerase-immortalized human epithelial cell line. We show for the first time that PDCD4 is required for the G1/S-transition, demonstrating its crucial role in the cell cycle. Inhibition of p53-dependent activation of p21^WAF1/CIP1^ overrides the requirement for PDCD4 for the G1/S-transition, suggesting that PDCD4 counteracts basal p53 activity to prevent activation of the G1/S checkpoint by p53. Transcriptome and ribosome profiling data show that silencing of PDCD4 changes the expression levels and translation of many mRNAs, providing an unbiased view of the cellular processes that are affected by PDCD4 in an epithelial cell line. Our data identify PDCD4 as a key regulator of cell cycle- and DNA-related functions that are inhibited when it is silenced, suggesting that decreased expression of PDCD4 might contribute to tumor development by compromising genomic integrity.

## Introduction

The *PDCD4* (*Programmed cell death 4*) gene encodes a highly conserved nuclear-cytoplasmic shuttling protein that acts as a tumor suppressor (for recent reviews see refs. ^1,2^). PDCD4 contains two highly structured MA-3 domains located in the central and C-terminal parts of the protein, which mediate protein-protein-interactions with the translation initiation factor eIF4A. A putative unstructured domain at its N-terminal has been shown to mediate protein-protein- and protein-RNA-interactions^3–10^. PDCD4 was initially shown to suppress tumor development in an *in-vitro* mouse keratinocyte model of tumor promotion^11^, but has since been implicated as a tumor suppressor in a broad spectrum of human tumors^12–19^. Down-regulation of PDCD4 expression in tumor cells occurs by different mechanisms. *PDCD4* mRNA is targeted by several microRNAs, most prominently oncogenic microRNA miR-21, whose over-expression in cancer cells down-regulates *PDCD4* expression^20,21^. On the protein level, p70(S6K) kinase-mediated phosphorylation of PDCD4 triggers its ubiquitination by the E3 ubiquitin ligase complex SCF(βTRCP) and its subsequent degradation^22^. A large body of work has suggested that down-regulation of *PDCD4* expression contributes to tumor development by stimulating the mobility and the metastatic potential of tumor cells^18–20,23–25^. Furthermore, silencing of *PDCD4* has been shown to affect the cellular DNA-damage response, suggesting that decreased PDCD4 expression might compromise genomic stability and contribute to tumor development^26,27^.

PDCD4 has emerged as a critical regulator of protein translation due to its ability to interact with and inhibit the function of the eukaryotic translation-initiation factor eIF4A, a RNA helicase that promotes the unwinding of mRNA secondary structures present in the 5′-untranslated regions (UTRs) of certain mRNAs^3,4,19,28^. PDCD4 is therefore thought to suppress the cap-dependent translation of mRNAs with 5′-structured UTRs. This was supported by studies showing that PDCD4 suppresses the translation of RNAs containing engineered 5′-hairpin structures^3,4^ as well as by the identification of specific mRNAs regulated by this mechanism^19,28^. However, alternative mechanisms of translational suppression involving direct RNA-binding of PDCD4 to the coding regions of specific mRNAs have also been described^29,30^.

Our current understanding of the function of human PDCD4 derives mostly from work carried out with transformed tumor cells. Here, we have used a telomerase-immortalized human epithelial cell line to study the effect of PDCD4 silencing on the cell cycle, gene expression and mRNA translation. Our work reveals a novel role of PDCD4 in the regulation of the cell cycle and provides a more complete picture of its cellular functions.

## Results

### PDCD4 is required for the G1/S-transition in RPE cells

**Figure 1:**
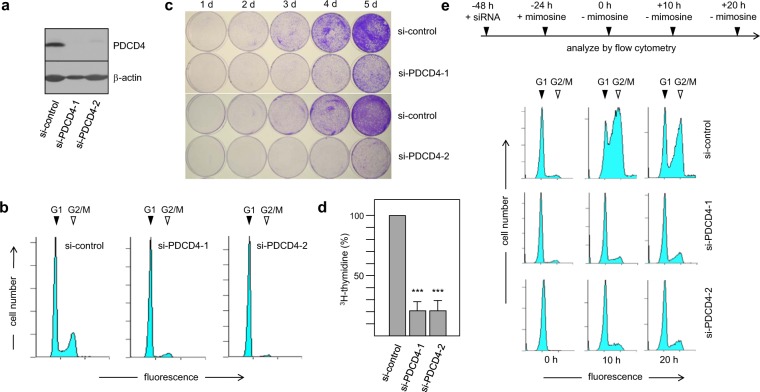
PDCD4 knockdown affects the cell cycle and growth properties of RPE cells. (**a**) Silencing of PDCD4 expression in RPE cells with PDCD4-specific siRNA-1 and -2. (**b)** Cell cycle distribution of RPE cells treated with control or PDCD4-specific siRNA-1 and -2. G1 and G2/M peaks are marked. (**c**) Equal numbers of RPE cells treated with control siRNA or PDCD4 siRNA-1 or -2 were plated onto replicate tissue culture plates. The growth of the cells was followed over several days by fixing one of the replicate plates at each indicated day of culture with formaldehyde. After 5 days of culture all plates were stained simultaneously with crystal violet. (**d**) RPE cells treated with siRNAs as in A. The cells were then incubated in medium supplemented with 10 μCi/ml 3H-thymidine for 1 hour. Subsequently, the radioactivity incorporated into DNA was determined by TCA-precipitation and liquid scintillation counting. The bars indicate the percentage of DNA synthesis (with standard deviation) of the PDCD4 siRNA treated cells relative to control cells. Asterisks indicate statistical significance (**p < 0.01; ***p < 0.001; students-t test). (**e)** RPE cells were treated for 24 h with control siRNA or PDCD4-specific siRNA-1 and -2. The cells were then arrested in the late G1 phase by incubation for 24 hours in the presence of 0.5 mM mimosine. Cells were then processed immediately for flow cytometry analysis or were washed with fresh medium lacking mimosine and cultivated for additional 10 or 20 hours before being analyzed by flow cytometry. G1 and G2/M peaks are marked.

Our current understanding of PDCD4′s role in human cells is largely based on studies using transformed tumor cell lines. Such studies have provided insight into the function of PDCD4 as a tumor suppressor but may not reveal an unbiased picture of its cellular roles due to the aberrant nature of these cells. To study the function of human PDCD4 in normal cells we have used the telomerase-immortalized hTERT-RPE-1 cell line (referred to as RPE hereafter) as a model of untransformed epithelial cells. Expression of PDCD4 was effectively silenced by two different siRNAs (Fig. 1a). The cells did not show obvious changes of their spindle-shaped fibroblast-like morphology when viewed under the microscope. To explore whether PDCD4 knockdown disrupts the cell cycle we examined the cell cycle distribution of asynchronous cultures of RPE cells treated with PDCD4-specific or control siRNAs by flow cytometry. The cell cycle profiles of the control and PDCD4 knock-down cells were different. Specifically, the abundance of S- and G2-phase cells was strongly decreased in cultures treated with the two different PDCD4-specific siRNAs compared to the control cells (Fig. 1b and Supplementary Table S1). Both siRNAs yielded similar results suggesting that the partial G1 arrest is induced by PDCD4 knockdown and not by off-target effects.

Based on this observation we hypothesized that PDCD4 knockdown decreases the proliferation rate of the cells. To test whether this is the case, we monitored the growth of the cells over a period of 5 days following knockdown with PDCD4-specific or control siRNA. We used a qualitative assay of cell proliferation by plating equal numbers of cells on replicate culture plates and visualized their proliferation by crystal violet staining (Fig. 1c). We found that the intensity of staining of cells transfected with PDCD4-specific siRNAs was decreased compared to the control cells, suggesting decreased proliferation upon Pdcd4 knockdown. As in the previous experiment, both Pdcd4-specific siRNAs had similar effects.

To substantiate the finding that PDCD4 knockdown reduces the proliferation of RPE cells we quantified their DNA-synthesis activity as an independent measure of proliferation. We performed ^3^H-thymidine incorporation assays by incubating equal numbers of control- and PDCD4-knockdown cells for 1–2 hours in the presence of radiolabeled thymidine and determined the amount of radioactivity incorporated into TCA-precipitable high molecular weight DNA. RPE cells treated with the PDCD4-specific siRNAs displayed significantly reduced DNA synthesis activity compared to cells treated with control siRNA (Fig. 1d). Thus, in comparison to the control cells the number of cells in S-phase was significantly reduced in cultures treated with PDCD4-specific siRNA. This is consistent with our cell cycle measurements where almost no S-phase cells were visible after PDCD4 knockdown (Fig. 1b).

Overall, these experiments suggested a slowed-down entry of cells into S-phase when PDCD4 levels are low. To demonstrate the effect of PDCD4 knockdown on the G1/S-transition more directly we silenced PDCD4 expression and synchronized the cell population by an additional treatment for 24 hours with 0.5 mM mimosine to block DNA-replication^31^. This leads to a reversible arrest of most of the cells at the G1/S boundary. The cells were then released into S-phase by washing them with medium without mimosine, followed by an analysis of the cell cycle profile immediately after the release of the cell cycle block and after 10 and 20 hours (Fig. 1e and Supplementary Table S1). Most of the G1-arrested, control siRNA-treated cells had entered into S-phase within 10 hours after removal of mimosine. At 20 hours there was a distinct G2-peak and an increased G1 peak, suggesting that a fraction of the cells had progressed through mitosis to reach the subsequent G1-phase. In contrast, only a small fraction of the cells treated with either of the PDCD4-specific siRNAs had entered into S-phase even after 20 hours, indicating that PDCD4 knockdown had strongly blocked the G1/S-transition.

### Defective G1/S-checkpoint control overrides the requirement for PDCD4 to undergo G1/S-transition

**Figure 2:**
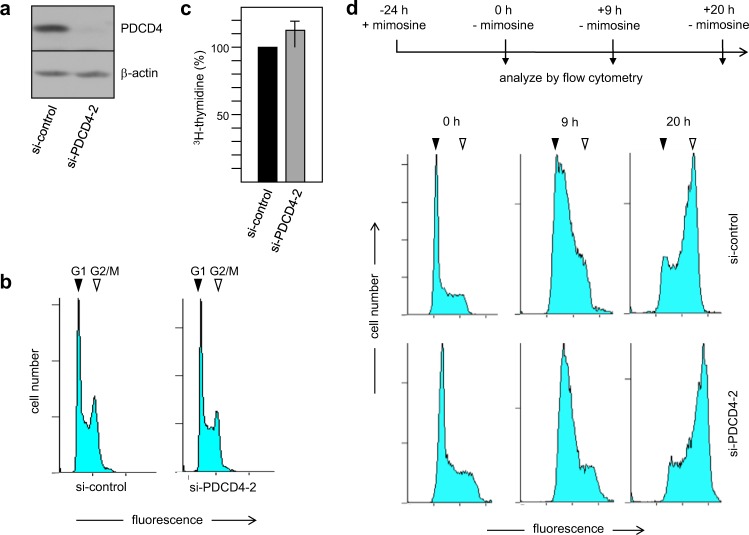
The G1/S-transition is independent of PDCD4 expression in HEK293T cells. (**a**) Silencing of Pdcd4 expression by treatment of HEK293T cells with PDCD4-siRNA-2. (**b**) Cell cycle distribution of HEK293T cells treated with control or Pdcd4-specific siRNA-2. G1 and G2/M peaks are marked. (**c)** HEK293T cells treated for 72 hours with control or PDCD4-specific siRNA as in A were incubated for 1 h in medium supplemented with 10 μCi/mL ^3^H-thymidine. The radioactivity incorporated into DNA was determined by TCA-precipitation and liquid scintillation counting. The bars indicate the percent DNA synthesis (with standard deviation) of the PDCD4 siRNA treated cells relative to control cells. (**d)** HEK293T cells treated for 24 hours with control or PDCD4-specific siRNA were analysed for the G1/S-transition as in Fig. 1e. Positions of the G1 and G2 peaks are marked.

The finding that PDCD4 is required for the G1/S-transition seems counterintuitive considering that PDCD4 expression is often decreased in tumor cells^1,2^. We therefore hypothesized that defective G1/S-checkpoint control, which is a hallmark of many tumor cells, might circumvent the requirement for PDCD4 to pass the G1/S-boundary. To test whether defective G1/S-checkpoint control can override the requirement for PDCD4 for cells to enter into S-phase we investigated the effect of PDCD4 knockdown in HEK293T cells. These cells express the adenoviral E1A protein and the SV40 large T antigen, both of which sequester the retinoblastoma protein and allow transcription factor E2F to be active independently of cyclin/Cdk-induced phosphorylation, thereby inactivating the G1/S-checkpoint^32,33^. Although PDCD4 expression was silenced effectively in these cells (Fig. 2a) the cell cycle distribution of an asynchronous culture of HEK293T cells is almost indistinguishable, as judged by the slightly reduced height of the G2-cell peak and the almost similar height of the plateau of S-phase cells between the G1 and G2 peaks when comparing the control and the Pdcd4-knockdown cell populations (Fig. 2b and Supplementary Table S1). Moreover, we observed no significant change in the incorporation of ^3^H-thymidine into DNA (Fig. 2c). Furthermore, we used mimosine to block HEK293T cells in the cell cycle. Because mimosine arrests cells at the G1/S-boundary as well as cells that have already entered S-phase, mimosine-treatment of HEK293T cells resulted in a peak of G1-cells with a pronounced shoulder towards a higher DNA-content. Importantly, the cell cycle profiles recorded at 9 and 20 hours after removal of mimosine showed that PDCD4-silenced HEK293T cells had entered into S-phase upon removal of the drug similar to the control cells (Fig. 2d and Supplementary Table S1). This indicates that PDCD4 knockdown does not affect the G1/S-transition in HEK293T cells.

### Knockdown of PDCD4 activates the G1/S-checkpoint in RPE cells by increasing the expression of p21^WAF1/CIP1^

**Figure 3:**
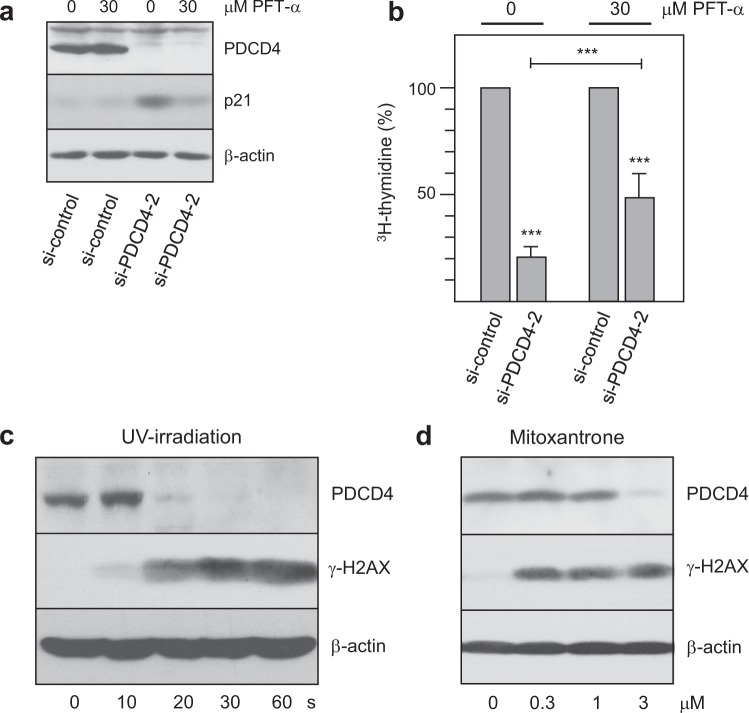
PDCD4 is linked to the G1/S checkpoint via the p53-p21^WAF1/CIP1^ axis. (**a)** Knockdown of PDCD4 increases the expression of p21^WAF1/CIP1^. RPE cells were transfected with control siRNA or PDCD4-specific siRNA-2 in the absence or presence of 30 μM PFT-α. Total cell extracts were then analyzed by western blotting for expression of PDCD4, p21^WAF1/CIP1^ and β-actin. (**b)** RPE cells were labeled with ^3^H-thymidine for 1 hour, followed by TCA precipitation and liquid scintillation counting to determine their DNA synthesis activity. The bars indicate the percentage of DNA synthesis (with standard deviation) of the PDCD4 siRNA treated cells relative to control cells. Asterisks indicate statistical significance (***p < 0.001; students-t test). (**c,d)** DNA damage induced down-regulation of PDCD4 expression. RPE cells were exposed to UV light for the indicated times, using a germicidal UV-C lamp in a tissue culture hood or were cultivated in the presence of the indicated concentrations of mitoxantrone. Cells were incubated for 16 hours and total cell extracts were analyzed by western blotting for expression of PDCD4, the DNA double strand break marker γ-H2AX, and β-actin.

Taken together, our data support the concept that PDCD4 knockdown activates the G1/S cell cycle checkpoint in RPE cells, thereby delaying cell cycle progression at the G1/S boundary. We have previously reported that knockdown of PDCD4 increases the activity and expression of p53 and thereby stimulates the expression of the p53 target gene *CDKN1A*^26,28^. *CDKN1A* encodes the Cdk-inhibitor p21^WAF1/CIP1^ that plays a key role at the G1/S-checkpoint. DNA damage induces p21^WAF1/CIP1^ expression via p53, which then inhibits Cdk activity and causes a G1/S cell cycle arrest^34,35^. Therefore, we hypothesized that the requirement for PDCD4 to enter the S-phase was due to its ability to balance or counteract the basal activity of p53 in unstressed cells. This would suggest that decreasing the activity of p53 by an inhibitor would relieve the requirement for PDCD4 expression for S-phase entry. To test this possibility, we employed pifithrin-α (PFT-α), an inhibitor that suppresses the p53-dependent activation of p53 target genes^36^. We knocked down PDCD4 both in the presence or absence of PFT-α and analyzed the expression of p21^WAF1/CIP1^ by western blot. Interestingly, we found a strong increase of p21^WAF1/CIP1^ expression following knockdown of PDCD4 in the absence of PFT-α (Fig. 3a), consistent with our earlier studies^26^ and demonstrating that silencing of PDCD4 increases p21^WAF1/CIP1^ expression also in RPE cells. As expected, in the presence of 30 μM PFT-α the increase of p21^WAF1/CIP1^ expression was strongly suppressed. To measure S-phase entry of the cells we performed ^3^H-thymidine-labeling experiments. This showed that knockdown of PDCD4 in the absence of PFT-α strongly reduced DNA-synthesis activity (Fig. 3b), whereas the inhibition of DNA-synthesis activity by PDCD4 knockdown was less strong in the presence of PFT-α. It is possible that the DNA synthesis activity of PDCD4 knockdown cells did not reach the level of control cells in the presence of PFT-α because there was still a residual increase of p21^WAF1/CIP1^ in the presence of PFT-α. This could be due to a limiting concentration of the inhibitor, or could reflect a minor contribution of a p53-independent mechanism of stimulation of p21^WAF1/CIP1^ expression by PDCD4 silencing, as proposed recently^37^. Overall, our data show that the G1/S-cell cycle block induced by PDCD4 knock-down is caused, at least to a significant part, by the p53-dependent increase of p21^WAF1/CIP1^ expression. PDCD4, therefore, plays a crucial role in counteracting basal p53 activity in unstressed cells.

Previously, we had observed that DNA damage down-regulates PDCD4 expression in HepG2 cells, suggesting a role of PDCD4 in the DNA-damage response^28^. This prompted us to examine whether the expression of PDCD4 is also decreased in response to DNA damage in RPE cells. We employed UV-irradiation and the topoisomerase inhibitor mitoxantrone to induce DNA-damage, which we monitored by the DNA double strand break marker γ-H2AX^38^. The increase of g-H2AX staining between untreated cells (first lanes in panels C and D) and cells UV-irradiated or incubated with mitoxantrone confirmed that both treatments caused DNA damage, which was accompanied by virtually complete loss of PDCD4 expression (Fig. 3c,d). This suggested that PDCD4 does not act as an antagonist of p53 in the presence of genotoxic stress.

### Silencing of PDCD4 in RPE cells affects the abundance and translation of multiple mRNAs

**Figure 4:**
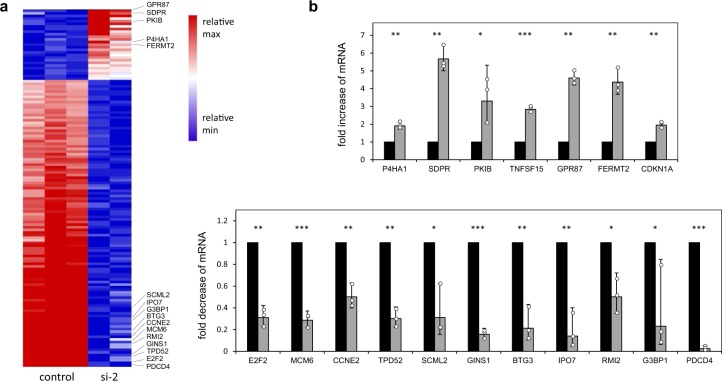
Pdcd4 knockdown induces transcriptome-wide changes of mRNA expression. (**a**) Heat map of all mRNAs whose expression levels were significantly altered (padj value < 0.05) after silencing of PDCD4 by a log2-fold change > 0.5 or <−0.5. The individual columns represent the results of three independent samples from cells transfected with control siRNA (control) and two independent samples of cells transfected with PDCD4 siRNA-2 (si-2). Selected genes are marked on the right side. (**b**) Real-time PCR analysis of selected up- and down-regulated RNAs. The columns indicate mRNA abundance in control siRNA (black bars) and PDCD4 siRNA-2 (grey bars) treated RPE cells. Individual expression levels determined from three independent biological replicates are marked by white dots. Asterisks indicate statistical significance (*p < 0.05; **p < 0.01; ***p < 0.001; students-t test).

To obtain an integrated view of the functions of PDCD4, we employed RNA-Seq and ribosome profiling. This allowed us to explore the effect of PDCD4 knockdown in RPE cells on transcriptome-wide mRNA abundance and translation (Supplementary Fig. 1a). First, we used PDCD4 siRNA-2 and control siRNA in RPE cells (Supplementary Fig. 1b) and subjected them to RNA-Seq. We found that 496 genes were significantly upregulated and 750 genes were down-regulated by PDCD4 silencing. The heat map (Fig. 4a) shows all genes that are up- or down-regulated two-fold or more. We validated these changes by quantitative real-time PCR to confirm the expression of representative up- and down-regulated mRNAs (Fig. 4b).

**Figure 5:**
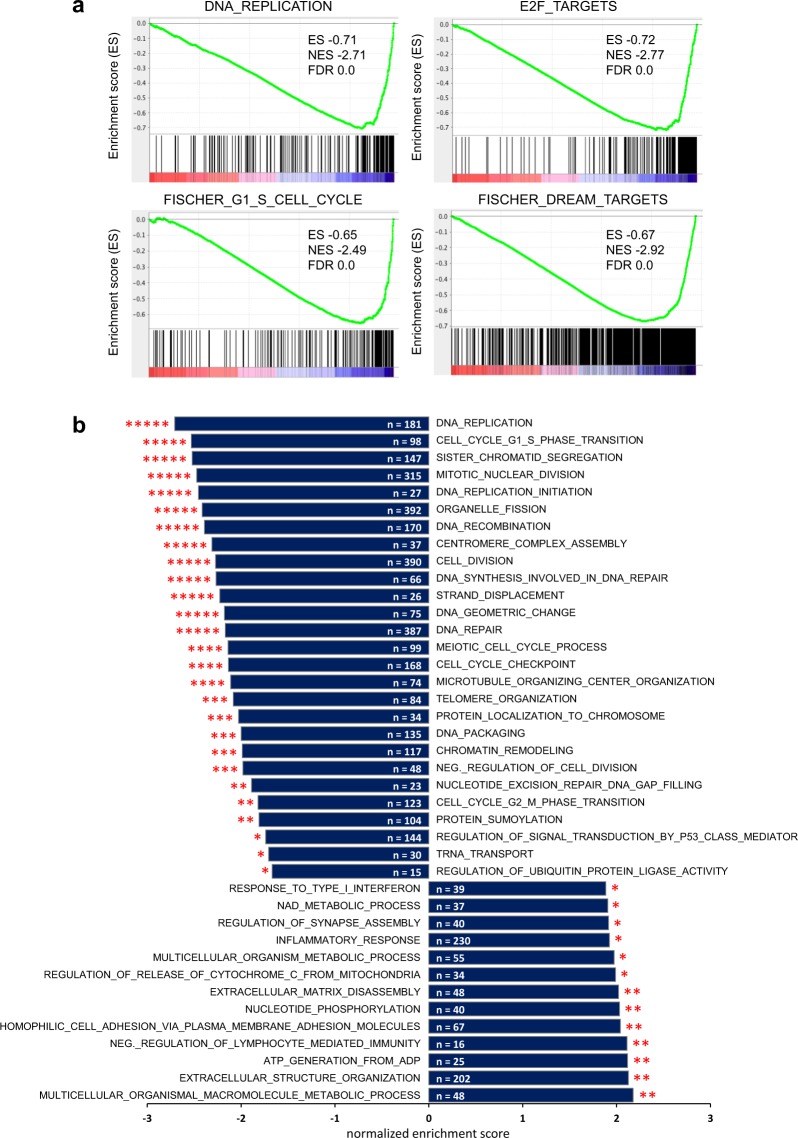
Gene set enrichment analysis of genes affected by PDCD4 knockdown. (**a)** Examples of GSEA charts revealing the role of PDCD4 in cell cycle regulation. (**b)** GO-term (biological process) enrichment analysis of genes up- and downregulated by PDCD4 silencing. Significantly differentially expressed gene sets were identified by GSEA and reduced to most significant terms by REVIGO. Positive and negative enrichment scores, respectively, indicate up- and down-regulation in PDCD4 knockdown cells. Numbers indicate counts of genes in each gene set. The asterisks indicate FDR q-values (*<0.05; **<0.01; ***<0.001; ****<0.0001; ***** < 0.00001).

To investigate whether PDCD4-dependent changes of mRNA expression affect specific biological processes we performed gene set enrichment analysis (GSEA^39^). mRNAs suppressed by PDCD4 silencing were strongly enriched in genes that are implicated in DNA replication, E2F targets and cell cycle regulation (Fig. 5a and Supplementary Table S2), substantiating our previous findings. Genes bound by the DREAM complex, a transcriptional regulatory complex playing a key role in cell cycle regulation^40–42^, and genes with a peak of expression at the G1/S-checkpoint were strongly downregulated upon PDCD4 silencing. Further gene ontology (GO) term analysis of genes repressed by PDCD4 knockdown confirmed that these genes were involved in various DNA-related processes and aspects of cell cycle regulation. Genes that were up-regulated by PDCD4 knockdown were enriched in processes related to immune responses, aspects of extracellular matrix organization, cytokine signalling and motility (Fig. 5b). Overall, these findings suggest that decreased expression of PDCD4, as seen in many tumor cells, similarly affects a plethora of cellular processes. Furthermore, the data underline the notion that PDCD4 plays a crucial role in cell cycle regulation, particularly at the G1/S-phase transition and the subsequent S-phase.

**Figure 6:**
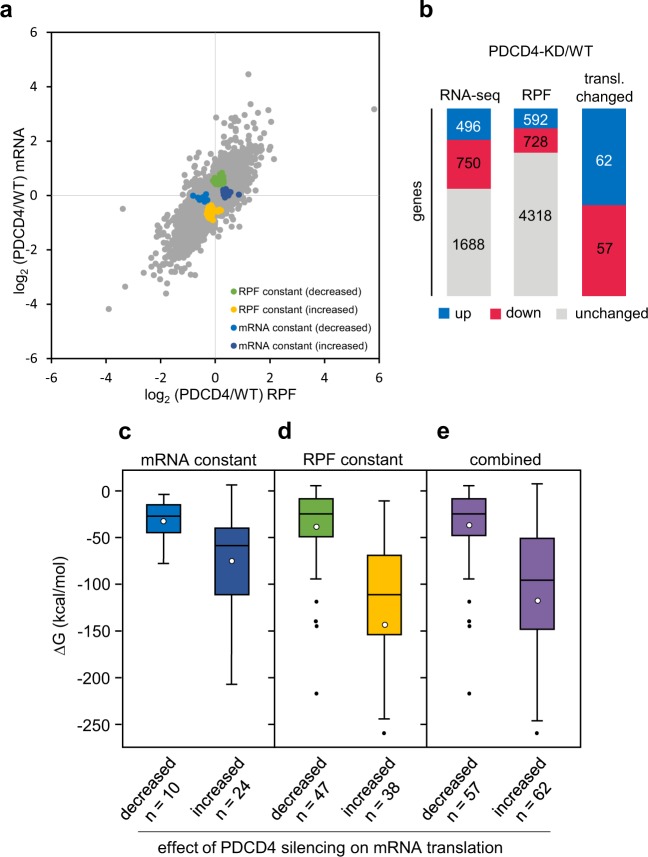
PDCD4 silencing affects the translational landscape of RPE cells. (**a)** Scatter plot showing the log_2_-fold changes on the mRNA (y-axis) and ribosome footprint (x-axis) levels in PDCD4 knockout RPE cells relative to control knockout cells. Genes altered only in one parameter by PDCD4 silencing are represented as colored dots (translationally changed genes in B). (**b)** Changes in RNA-Seq, RPF, and translationally changed genes between control and PDCD4 knockout cells. For RNA-seq and RPF data, “up” and “down” includes genes with a log_2_-fold change > 0 or <0, respectively, and an adjusted p-value < 0.05. For translationally changed genes, “up” and “down” includes genes with an adjusted p-value < 0.05 for mRNA or RPF and a corresponding adjusted p-value for unchanged hypothesis testing < 0.1. **(c–e)** Box-plots showing the distribution of ΔG values for RNA secondary structures in the 5′-UTRs of mRNAs translationally suppressed or activated by PDCD4 silencing.

To assess the global effects of PDCD4 on mRNA translation we performed ribosome profiling^43,44^ using RPE cells transfected with PDCD4-specific siRNA-2 or control siRNA. In this approach, polysomes of control and PDCD4 knockdown RPE cells are nuclease digested, ribosome-protected mRNA fragments (RPF) isolated and used for deep sequencing to generate “snapshots” of global translation. By combining these data with RNA-Seq data it is possible to determine the effect of PDCD4 silencing on the translation efficiency of individual mRNAs (Fig. 6a). By comparing the RNA-Seq and ribosome profiling data from control and PDCD4 knockdown cells we identified 496 transcripts that were significantly induced and 750 genes that were downregulated, while 1688 genes remained unchanged by PDCD4 silencing (“RNA-Seq” in Fig. 6b). The RPF analysis showed that 592 and 728 genes had increased or decreased RPF levels, while 4388 genes did not exhibit significant translational changes upon PDCD4 silencing (“RFP” in Fig. 6b). For the majority of transcripts the changes in translation levels correlate with altered mRNA abundance following PDCD4 silencing. mRNAs that are translationally regulated by PDCD4 knockdown (“translationally changed” in Fig. 6b) were defined as transcripts that exhibit altered RPF levels, while mRNA levels remained unaffected in response toPDCD4 silencing. This resulted in the identification of 34 mRNAs (Supplementary Table S3). Since PDCD4 has been implicated in the translational suppression of mRNAs with structured 5′-UTRs^3,4^, we used mfold^45^ to examine the potential of the 5′-UTRs of the selected mRNAs to form secondary structures. We plotted the ΔG-values predicted for the folding of the 5’-UTRs separately for those mRNAs that showed increased or decreased translation after PDCD4 knockdown (Fig. 6c and Supplementary Table S3). This indicated that mRNAs whose translation was increased by PDCD4 silencing had more negative predicted ΔG-values, reflecting a higher secondary structure potential of their 5′-UTRs, than those mRNAs whose translation was decreased by the PDCD4 knockdown. We further identified 85 mRNAs that remained stable at the level of translation but were altered at the mRNA levels upon PDCD4 silencing and, hence, were also differentially translated between control and PDCD4 knockdown cells (Supplementary Table S3). We determined the predicted ΔG-values for folding of the 5′-UTRs of the selected mRNAs whose translation was increased or decreased by PDCD4 silencing (Fig. 6d and Supplementary Table S3). Similar to our previous analysis this showed that mRNAs whose translation was more effective after PDCD4 knockdown have lower ΔG-values for folding of their 5′-UTRs than mRNAs whose translation was decreased. Combining these different sets of mRNAs, we identified 62 translationally up-regulated and 57 down-regulated mRNAs upon PDCD4 silencing (“translationally changed” in Fig. 6b), of which mRNAs with increased translation after knockdown of PDCD4 possess more highly structured 5′-UTRs than mRNAs whose translation is decreased when PDCD4 is silenced. (Fig. 6e). Although we cannot exclude indirect effects of PDCD4 silencing on the translation of specific mRNAs, our analyses are consistent with the concept that PDCD4 suppresses the translation of mRNAs that contain structured 5′-UTRs. Besides the identification of mRNAs that are potential targets of translational suppression by PDCD4, our work has also revealed mRNAs that show decreased translation upon PDCD4 knockdown. The identification of groups of mRNAs whose translation is either positively or negatively regulated by PDCD4 sets the stage for future work to understand the role of PDCD4 in translation regulation in more detail.

## Discussion

PDCD4 is a multifunctional protein initially described as a transformation suppressor in a murine keratinocyte transformation model^11^. Subsequent work has strongly suggested that PDCD4 acts as a tumor suppressor in a broad spectrum of human tumor types^1,2^ and has shown that decreased expression of human PDCD4 contributes to tumor development in various ways, for example by enhancing the motility and invasiveness of the tumor cells^18–20,23–25^. Most of the studies addressing the function of human PDCD4 have employed various tumor cells, raising the question whether these studies fully reflect the function of human PDCD4 in normal cells. Therefore, we have used a telomerase-immortalized human epithelial cell line to highlight novel aspects of PDCD4′s function.

Our work shows for the first time that PDCD4 is required for the G1/S-phase transition. We observed that siRNA-mediated down-regulation of PDCD4 expression strongly impaired the entry of the cells into S-phase, decreased DNA synthesis activity and reduced cell proliferation rate. Our results suggest that the role of PDCD4 as a G1/S cell cycle regulator is linked to the activity of p53. More specifically, our work supports the notion that PDCD4 is required to counteract the activity of p53, preventing the activation of the G1/S-checkpoint in unstressed cells and permitting them to enter into S-phase. In this scenario, knockdown of PDCD4 leads to increased p53-dependent expression of p21^WAF1/CIP1^ and concomitant activation of the G1/S-checkpoint. Using HeLa cells, we have previously observed increased p21^WAF1/CIP1^ expression after knockdown of PDCD4^26^. However, unlike the work reported here, knockdown of PDCD4 in HeLa cells only showed aberrant cell behaviour in the presence of DNA damage but did not result in overt cell cycle defects. This might be due to the defective nature of the G1/S-checkpoint in these cells caused by the sequestration of the RB protein by the human papilloma virus E7 protein expressed in HeLa cells^46^. HEK293T cells also have a defective G1/S checkpoint (resulting from the expression of the adenovirus E1A and E1B proteins). We found that the requirement for PDCD4 expression for S-phase transition is indeed absent in these cells. Overall, our work identifies a novel role of PDCD4 as a cell cycle regulator that balances p53 activity in unstressed cells, presumably to prevent G1/S-checkpoint activation. Interestingly, we also showed before^28^ and confirmed here that induction of DNA damage leads to down-regulation of PDCD4 expression, which suggests that this function of PDCD4 is abolished under conditions of genotoxic stress.

The identification of a pro-proliferative role for PDCD4 in the cell cycle is somewhat unexpected in the light of its function as a tumor suppressor. At first glance, low expression of PDCD4 in tumor cells would be expected to impede the cell cycle, however, many tumor cells have a defective G1/S checkpoint as a result of p53 or other mutations, neutralizing the inhibitory effects of low PDCD4 expression on the G1/S-transition. Transcription profiling has revealed a large number of genes that are up- or down-regulated upon PDCD4 knockdown, providing an unbiased view of the cellular processes that are affected by PDCD4. Consistent with previous studies showing increased motility and invasiveness^18–20,23–25^, GO-term analysis for the biological function of the genes up-regulated by PDCD4 knockdown identifies functions related to extracellular matrix organization and cell adhesion amongst others. GO-term analysis of genes down-regulated by PDCD4 knockdown identifies a plethora of cell cycle- and DNA-related functions that are inhibited when PDCD4 expression is low, such as DNA-replication, DNA-recombination, DNA-repair, telomere organization, chromosome segregation and chromatin remodelling, amongst others. This suggests that decreased PDCD4 expression contributes to tumor development and progression by compromising genomic integrity.

Finally, our ribosome profiling analysis shows that the translation of the majority of transcripts was not affected by silencing of PDCD4 because changes in the abundance of ribosome footprints correlated with changes in the expression levels of these mRNAs. By focussing on transcripts that were affected in only one parameter (i.e. mRNA expression level or the frequency of RPF reads) in response to PDCD4 knockdown, we have identified several mRNAs whose translation was moderately increased following PDCD4 knockdown, suggesting that they might be translational targets of PDCD4. These RNAs exhibit an increased potential to form stable secondary structures in their 5′-UTRs compared to mRNAs showing decreased translation after PDCD4 silencing, consistent with the notion that Pdcd4 preferentially inhibits translation of RNAs with structured 5′-UTRs^3,4^. Similarly, PDCD4 knockdown stimulates the translation of RNAs that lack secondary structure in their 5′-UTR or that have short 5′-UTRs. Whether the translation of these RNAs is suppressed by binding of PDCD4 to their coding regions, as already reported for certain mRNAs^29,30,47^, or whether PDCD4 affects their translation indirectly remains to be addressed by future studies. In addition to the identification of potential target mRNAs for translational repression by PDCD4 we have also discovered mRNAs whose translation is positively affected by PDCD4. Whether PDCD4 mediates these effects directly or indirectly and whether this reflects a novel aspect of the function of PDCD4 in translation, remains to be investigated in future work. Overall, our study is the first analysis of genome-wide changes of mRNA abundance and translation induced by PDCD4 silencing in an immortalized human epithelial cell line. This sets the stage for more detailed studies on the role of PDCD4 in the future.

## Materials and Methods

### Cells and siRNA transfections

hTERT-RPE-1 is a line of telomerase-immortalized human retina pigment epithelial cells^48^. The cells were grown in DMEM/Ham’s F12 medium supplemented with 10% fetal calf serum. PDCD4 expression was silenced with siRNA duplexes targeting the sequences CACCAAUCAUACAGGAAUA (PDCD4 siRNA-1) or GCUUCUUUCUGACCUUUGU (PDCD4 siRNA-2). SiRNA targeting Renilla luciferase (AAACAUGCAGAAAAUGCUG) was used as negative control. siRNAs (100 nM) were reversely transfected using Lipofectamine^®^ RNAiMax (ThermoScientific), according to manufacturer’s protocols. Cells were harvested 48 to 72 h after transfection.

### Cell cycle analysis

Cells were trypsinized, fixed with 70% ice-cold ethanol in PBS for 1 h or longer at −20 °C, washed with PBS (+0.5% BSA) and stained with propidium iodide (50 μg/mL PI and 25 μg/mL RNase A in PBS) for 1 h at room temperature. In some experiments, cells were synchronized by incubation for 24 h in growth medium containing 0.5 mM mimosine. To release the cells into the cell cycle they were washed twice with grown medium lacking mimosine. Flow cytometry analysis was performed using a Beckman-Coulter Cytomics FC500 flow cytometer. 10 000 to 15 000 cells were counted per condition in every experiment.

#### Antibodies

Western blotting of PDCD4 was performed using a rabbit anti PDCD4 antiserum raised against the N-terminus of human Pdcd4^26^. Antibodies against p21^WAF1/CIP1^ (05–345, Millipore), γ-H2AX (GTX61796, Genetex) and β-actin (AC15, Sigma-Aldrich) were obtained from commercial sources.

#### Quantitative real-time PCR

Total cellular RNA was isolated with TRIzol^TM^ Reagent (Invitrogen), as recommended by the manufacturer. Total RNA (2 μg) was reverse transcribed with the First Strand cDNA Synthesis Kit (K1612, ThermoScientific) using OligoT primers in 20 μL according to the manufacturer’s instructions. Real-time RT-PCR reactions were carried out in 96-well plates using Power SYBR Green PCR Master Mix (Applied Biosystems). Reactions were performed using a StepOnePlus RT-PCR instrument (Applied Biosystems) and the following parameters: 95 °C for 10 min, followed by 40 cycles of 95 °C for 15 s and 60 °C for 60 s. Each experiment included a no-template control. PCR reaction specificity was confirmed by melting curve analysis of the products. Primer sequences are given in Supplementary Table S4. Relative gene expression was calculated by the ΔΔC_T_ method:^49^ First, ΔC_T_ values were calculated by subtracting the C_T_-values obtained for individual mRNAs from those obtained for β-actin mRNA. Then, ΔΔC_T_ values were calculated by subtracting the ΔC_T_ values of Pdcd4 siRNA-treated cells from those of control siRNA-treated cells. All experiments were conducted with at least three biological replicates.

### ^3^H-thymidine labeling

Cells were incubated with growth medium supplemented with 10 μCi/ml 3H-thymidine for 1 h. The cells were then washed with PBS, lysed in PBS containing 1% SDS and heated to 95 °C to reduce the viscosity. Aliquots were then spotted on Whatman filter paper and washed 2 times for 15 min with 10% trichloracetic acid (TCA) and once with ethanol. The filter paper was dried and the radioactivity was determined in a scintillation counter. To correct for differences in the cell number between Pdcd4-specific and control knockdown samples aliquots of the lysed cells were spotted on nitrocellulose membrane and hybridized to a ^32^P-labeled probe of total human DNA. Alternatively, aliquots of the lysed cells were analyzed by SDS-PAGE and western blotting for expression of β-actin to determine the relative number of cells.

### RNA-seq

For RNA-seq of poly(A)-selected RNA, RPE cells were incubated for 24 h after transfection with siRNA (Pdcd4 siRNA-2 or control siRNA), and cells were directly lysed in TRIzol^TM^ reagent (Invitrogen). Total RNA was extracted with 1-bromo-3-chloropropane, precipitated with EtOH, resuspended in milliQ water and treated with TURBO DNase (Ambion) for 30 min at 37 °C, 1400 rpm. RNA was extracted again with acidic phenol to remove DNase. The quality of the RNA was examined with an Agilent Bioanalyzer. Sequencing libraries of poly(A)-enriched RNA were finally generated with the TruSeq Stranded mRNA LT Kit (Illumina).

### Ribosome profiling

Ribosome profiling was carried out as previously described^44^. RPE cells were incubated for 24 h after transfection with siRNA (PDCD4 siRNA-2 or control siRNA). 2 h before harvesting the culture medium was replaced with fresh medium. To stabilize elongating ribosomes, cells were treated with 100 µg/mL cycloheximide (CHX) for 5 min at 37 °C, following a washing step with ice cold PBS (containing 100 µg/mL CHX). Cells were then lysed in lysis buffer (10 mM Tris-HCl (pH 7.4), 10 mM MgCl_2_, 100 mM NaCl, 1% Triton, 1 mM DTT, 100 µg/mL CHX) per sample. Each sample consisted of cells from 8 tissue culture dishes (10 cm diameter), and cell debris was pelleted at 4 °C, 10 000 x g for 3 min. 10 OD_260_ units of cell extract were then supplemented with 900 U RNase I (Ambion) and 0.5% deoxycholate and treated for 20 min at 22 °C and 800 rpm in a thermomixer. The reaction was stopped by addition of 240 U SUPERase In (Ambion)( + 0.5% deoxycholate) and extracts were fractionated by centrifugation at 4 °C, 35 000 rpm for 3 h in a SW-41 Ti swinging-bucket rotor (Beckman Coulter) on 10–50% sucrose density gradients (20 mM Tris-HCl (pH 7.5), 10 mM MgCl_2_, 100 mM NH_4_Cl, 2 mM DTT, 100 µg/mL CHX). Gradients were fractionated at 0.75 mL/min with continuous monitoring of the OD_254_ using a Biocomp Instruments Gradient Station (Teledyne Isco). Monosome fractions were collected and, following addition of 1% SDS, flash-frozen and stored at −80 °C. RNA was isolated from gradient fractions by the hot acid phenol method (1-bromo-3-chloropropane used instead of chloroform), and ribosome footprints were purified from monosome RNA by size selection of 28–30 nt fragments (excluding a major band around 31 nt) on 15% polyacrylamide, 8 M urea, 1xTBE gels. Sequencing libraries from ribosome-protected footprints were generated by 3′-end dephosphorylation, followed by 3′-adapter ligation, reverse transcription, and circularization as described in^44^.

### Sequencing data analysis

The analysis of the ribosome profiling and RNA-seq datasets was essentially performed as described in^44^. Briefly, libraries for ribosome profiling and RNA-Seq were sequenced on an Illumina NextSeq sequencer. Ribosome-profiling reads were processed by clipping adapter sequences and trimming of the 4 randomized nucleotides in the linker with the FASTX-Toolkit version 0.0.13 (http://hannonlab.cshl.edu/fastx_toolkit). After processing, residual rRNA sequences were remove from ribosome-profiling datasets using bowtie version 1.0.0.^50^. Ribosome-profiling and raw RNA-Seq reads were mapped to hg38 transcripts (UCSC canonical transcripts extended 18 bp into the UTRs). Count tables of mapped reads were generated using custom scripts and differential expression determined with DESeq. 2^51^. Differential expression was scored using an adjusted p-value of 0.05 for the hypothesis of a changed gene (res) and unchanged expression was additionally scored using an adjusted p-value of 0.1 for the hypothesis of an unchanged gene (resLA). Transcripts translationally changed were defined with an adjusted p-value for mRNA or ribosome profiling data >0.05 (res) with the corresponding adjusted p-value in the other category (resLA) < 0.1.

### Gene set enrichment analysis (GSEA)

GSEA was carried out using the GSEA preranked tool (http://software.broadinstitute.org/gsea/index.jsp) with 1,000 gene set permutations. The genes in the expression dataset were ranked by their log_2_ fold change. Redundant terms were removed with REVIGO^52^.

### Calculation of ΔG values for 5′-UTR secondary structure formation

The 5′UTR sequences of the relevant mRNAs were retrieved from the NCBI nucleotide sequence data base and truncated immediately after the start codon. If several mRNA sequences were available sequence variant 1 was chosen. The sequences were then submitted to the RNAfold web server (http://rna.tbi.univie.ac.at) to predict the mimimum free energy of the optimal secondary structure (Supplementary Table S5).

### Data access

The RNA-seq and ribosome profiling data from hTERT-RPE-1 cells (PDCD4 siRNA2 and control siRNA) have been submitted to the NCBI Gene Expression Omnibus (GEO; www.ncbi.nlm.nih.gov/geo/) under accession number GSE138533 (https://www.ncbi.nlm.nih.gov/geo/query/acc.cgi?acc=GSE138533).

## Supplementary information

Supplementary data.

Supplementary tabel S2.

Supplementary tabel S3.
