# Supplementary material for: PDCD4 controls the G1/S-phase transition in a telomerase-immortalized epithelial cell line and affects the expression level and translation of multiple mRNAs
Source: Sci Rep. 2020 Feb 17;10:2758. doi: 10.1038/s41598-020-59678-w (PMC7026441; doi:10.1038/s41598-020-59678-w)

Supplementary information

PDCD4 controls the G1/S-phase transition in a telomerase-immortalized epithelial cell line and affects the expression level and translation of multiple mRNAs

Astrid Haas, Benedikt S. Nilges, Sebastian A. Leidel, Karl-Heinz Klempnauer

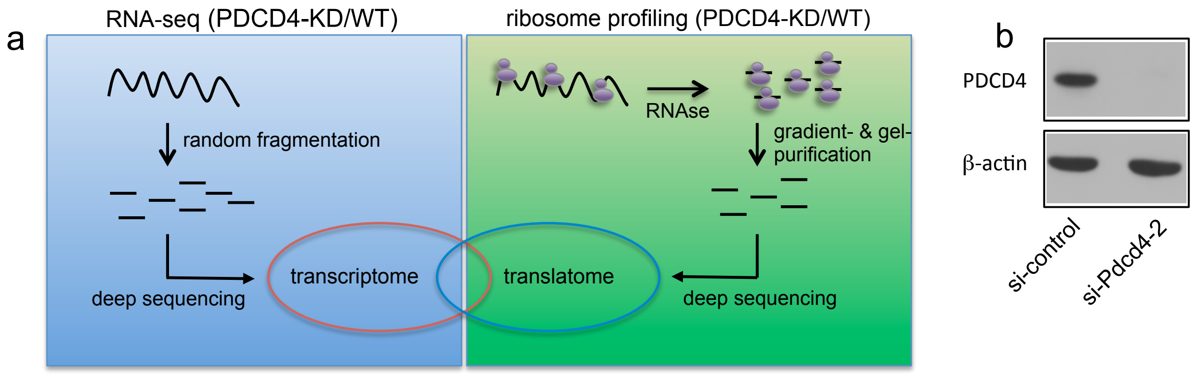

Supplementary Fig.1. **Pdcd4 knockdown induces transcriptome-wide changes of mRNA expression**. **a**. Schematic illustration of the experimental strategy. **b**. Western blot of RPE cells transfected with control siRNA or PDCD4-specific siRNA-2.

		G1	S	G2/M
Fig.1b	contr.-siRNA	47	27	26
	si-Pdcd4-1	88	5	7
	si-Pdcd4-2	93	2	4
		G1	S + G2/M	
Fig 1e	contr.-siRNA 0 h	86	13	
	contr.-siRNA 10 h	22	73	
	contr.-siRNA 20 h	33	60	
	si-Pdcd4-1 0 h	88	8	
	si-Pdcd4-1 10 h	71	20	
	si-Pdcd4-1 20 h	68	25	
	si-Pdcd4-2 0 h	82	17	
	si-Pdcd4-2 10 h	67	30	
	si-Pdcd4-2 20 h	69	20	
		G1	S	G2
Fig.2b	contr.-siRNA	46	29	23
	si-Pdcd4-2	49	29	20
		G1	S + G2/M	
Fig.2d	contr.-siRNA	64	36	
	contr.-siRNA	17	79	
	si-Pdcd4-2	55	45	
	si-Pdcd4-2	12	82	

Supplementary table S1. Percentage of cells in different cell cycle phases. The numbers refer to percent of the total population if cells that reside in the indicated cell cycle phases in the representative experiments shown in figures 1b, 1e, 2b and 2d.

Supplementary table S4: RT-PCR primer sequences

	primer	sequence
1	E2F2 RT-forward	ACCAACGTGCTGGAAGGCAT
2	E2F2 RT-reverse	ACATTCCCCTGCCTACCCACT
3	MCM6_fwd	CAGGCGAATCCTCGGCACTA
4	MCM6_rev	TCGCTGCTCTGAAACTCCTCC
5	CCNE2 RT-forward	GTAGCTGGTCTGGCGAGGTTT
6	CCNE2 RT-reverse	GGGCTGCTGCTTAGCTTGTA
7	TPD52 RT-forward	CCACAGAGACCCTCTCGGAA
8	TPD52 RT-reverse	CTGTCACGTCTTGCCACCCT
9	SCML2 RT-forward	ATCGCTGACCAGAGACCGTG
10	SCML2 RT-reverse	GGCGAAGCACCACATTCACC
11	GINS1_fwd	CCTGGCGCTGTAGGACTAGAA
12	GINS1_rev	GTCTGAGTCCATCCTCGTTGAA
13	BTG3 RT-foward	AGAGGCAGTTGAGAGGTTTGCT
14	BTG3 RT-reverse	CGAATACATCTGTAGGCCTGTCC
15	IPO7 RT-foward	GCCCTGCATATGATTGGCTCT
16	IPO7 RT-reverse	GTGAAGTACCCAGCAAGCCC
17	RMI2 RT-foward	AGTCCCAGGAAAGTATGTGATGGT
18	RMI2 RT-reverse	AATCTTCTACCTCCAGTTCCCACA
19	G3BP1 RT-foward	CTCAGCCGCGTAGGTTGAAT
20	G3BP1 RT-reverse	GACTGCATCTGCTGGCTTTCC
21	Pdcd4 RT-forward	ACATATCTGAAGCTGAACATTGCCT
22	Pdcd4 RT-reverse	ATGGTAGAAGACTTCCAAAGGGACT
23	P4HA1 RT-forward	GTACATGACCCTGAGACTGGAA
24	P4HA1 RT-reverse	AATGGGGTTCATACTGTCCTCC
25	SDPR RT-forward	TCCTCCGACGCAACCATTTC
26	SDPR RT-reverse	ATCTGAGGAGAGGTCCACGG
27	PKIB RT-forward	GCATCCCGGTGGACTGTAGA
28	PKIB RT-reverse	GTATAGCATGACTCCAGCCGC
29	TNFSF15 RT-forward	ATGCAACTCACAAAGGGCCGTC
30	TNFSF15 RT-reverse	CCAGGCCTAGTTCATGTTCCCA
31	GPR87 RT-forward	CACTTCCCTGCCGACCTTAGT
32	GPR87 RT-reverse	GGCGTGGGTTCAGCATAGGT
33	FERMT2_fwd	GCGACGACGGGACTCCATTA
34	FERMT2_rev	CCTTATCCCGTCCAGAGCCA
35	p21(CDKN1A)_fwd	CCTGTCACTGTCTTGTACCCT
36	p21(CDKN1A)_rev	GCGTTTGGAGTGGTAGAAATCT
37	ACTB_fwd	AGAGCTACGAGCTGCCTGAC
38	ACTB_rev	AGCACTGTGTTGGCGTACAG

Supplementary table S5: ΔG-calculations for 5'-UTRs

Nucleotide sequences used for ΔG calculations are listed belown. In case several mRNA variants for a given gene were available variant 1 was used.

RPF-changed; mRNA-unchanged (padj < 0.05 & resLA_padj < 0.1)

ATF7IP (Activating Transcription Factor 7 Interacting Protein)(4 transcript variants)

Variant 2 ΔG = -80.20 kcal/mol

```
  1 aatttgcata acggccccgg cgcgtgcaag ggagaagcgg gtttgttttt gaatctgcgg
 61 aggcggcggc ggtggcagcg gcggcgcggc gactgaagcg cgcgaaaagc tgaggcggca
121 acgtcgggga cggctgcgcg ggacggctct gtaggaagga acttggttcc ccctccctca
181 gcttccgccc caaaagattc aga
```

Variant 1 ΔG = -34.80 kcal/mol

```
  1 acacttttgt gcaactgaga ctgtgcagct gtctcaaaat ggctgctaac ctagtctttg
 61 tagctgctta ggcagcaagt atgtcagtga ggagacttgt cac
```

FASTK (Fas Activated Serine/Threonine Kinase)(5 transcripts variants)

Variant 1 ΔG = -13.40 kcal/mol

```
  1 atctaggggc tgctgggaag atggcggact cggtggctag ccg
```

ZFP36L1 (ZFP36 Ring Finger Protein Like 1)(3 transcript variants)

Variant 1 ΔG = -56.60 kcal/mol

```
  1 ggagtcagaa aggcgagggg cgccgggaac tggcgtgtgg gactccagac aggagaggct
 61 gcgccttccc cgcaccggga ccttcgcgac acaccagatc ctcgcccctg gctcgcgcga
121 acgcacagg
```

SLC25A1 (Solute Carrier Family 25 Member 1)(4 transcript variants)

Variant 1 ΔG = -21.00 kcal/mol

```
  1 gagcgcggag ttctggagtc tcggacccga agccgccaca gggcgccccg cctcccgccc
 61 gcc
```

ALDH16A1 (Aldehyde Dehydrogenase 16 Family Member A1)(2 transcript variants)

Variants 1 ΔG = -47.70 kcal/mol

```
  1 agccccgccc ctttgggctg gaaccggagg tgtcgctctt cggacctcaa ggttcccctt
 61 aacacagagc gccccgcagt cttcgcggaa agcgttcggg gtaggcg
```

CPSF1 (Cleavage And Polyadenylation Specific Factor 1)(1 transcript variant)

ΔG = -19.20 kcal/mol

```
 1 gagttcgctg ctgtcccggt tcctctcgag tcggctccaa ctgccagccc gggttggcgc
61 c
```

EMC1 (ER Membrane Protein Complex Subunit 1)(4 transcript variants)

Variant 1   ΔG = -2.90 kcal/mol

```
 1 gcatgcgctc gcatc
```

TMED10 (Transmembrane P24 Trafficking Protein 10/Transmembrane P24 Trafficking Protein 10)(1 transcript variant)

ΔG = -7.60 kcal/mol

```
 1 gaggccttcg gtggtgaacg agtctccagc acc
```

ACTN4 (Alpha-actinin-4)(2 transcript variants)

Variant 2   ΔG = -32.70 kcal/mol

```
 1 gaagcagctg aagcggcggt agcggcggcg gctcgggcag aggggcggga gctgaggcgg
61 gagcggacag gctggtgggc gagcgagagg cggcgga
```

Variant 1   ΔG = -32.70 kcal/mol

```
 1 gaagcagctg aagcggcggt agcggcggcg gctcgggcag aggggcggga gctgaggcgg
61 gagcggacag gctggtgggc gagcgagagg cggcgga
```

PLCB3 (1-phosphatidylinositol 4,5-bisphosphate phosphodiesterase beta-3/Phospholipase C Beta 3)(3 transcript variants)

Variant 1   ΔG = -77.30 kcal/mol

```
 1 agactggcgg gcgggcgggc actgacgccg cggggccgga gcgggccgcg cggtgggagc
61 agcggcgccg tcggtccccg tcagggctcc gtgggtcccc gacccgcccc tggccgggcc
```

CAPRIN1 (Cell Cycle Associated Protein 1)(2 transcript variants)

Variant 1   ΔG = -52.80 kcal/mol

```
  1 cactaggagc ggctctcggt gcagcgggac agggcgaagc ggcctgcgcc cacggagcgc
 61 gcgacactgc ccggaaggga ccaccacccct tgccccctca gctgcccact cgtgatttcc
121 agcggcctcc gcgcgcgcac g
```

PDIA6 (Protein Disulfide Isomerase Family A Member 6)(5 transcript variants)

Variant 4   ΔG = -44.50 kcal/mol

```
 1 gggacgtggg cgcgggggcg cggcgtgcgg cacgctgcag ggctgaagcg gcggcggcgg
61 tggggactgc acgtagcccg gcgctcggc
```

Variant 1   ΔG = -117.20 kcal/mol

```
 1 aaccgagggt gcccgctgca aggttaggtc tcctggcctc gtgcttcaaa caggaattaa
```

```
 61 tagaatccca ccaggtggtg tttgtgaggc gtagatggcg aggatatctg cagctgcagt
121 ttgctcattc tcacggctgc actgcattgc actgtgtgag cagaacacaa tttatccttc
181 cactgctgat gggcatttgg gcggtttgca gtttgggatc tggccagttc tgctgttgtg
241 agcattctta agcctgcctt tcggtgaatt cacacgcccg tggggacgct cactctcaga
301 gcacagcttc cagaggagag aagatacccc cagcatcc
```

PDIA3 (Protein Disulfide Isomerase Family A Member 3)(1 transcript variant)

ΔG = -35.50 kcal/mol

```
 1 agacgcgcga gcgcaagcag cgggttagtg gtcgcgcgcc cgacctccgc agtcccagcc
61 gagccgcgac ccttccggcc gtccccaccc cacctcgccg cc
```

ITGB1 (Integrin Subunit Beta 1)(5 (1A-1E) transcript variants)

Variant 1E ΔG = -82.60 kcal/mol

```
  1 gagccagccc agccgcgttc cgaacgtgag ggtcgccggc ctgggcgctg tcacgtcggg
 61 gctgccggag ctgcggggga ccgggcccga acggcccctg acacctgcgg tctcccgccg
121 ggctgggcaa gcgcag
```

Variant 1A ΔG = -40.30 kcal/mol

```
 1 gccagcccgc gggagaggcc cagcgggagt cgcggaacag caggcccgag cccaccgcgc
61 cgggccccgg acgccgcgcg gaaaag
```

LMAN1 (Lectin, Mannose Binding 1)(1 transcript variant)

ΔG = 2.20 kcal/mol

```
1 ctccgcgttc cagaatccaa g
```

TMED2 (Transmembrane P24 Trafficking Protein 2)(2 transcript variants)

Variant 1 ΔG = -47.90 kcal/mol

```
 1 gagaaggcag cggggcggcg gcggcggcgg cggcggcggc tgtggaggcc gcagtccggg
61 tcctggcttc ggcctcagcc ccacc
```

EIF2AK2 (Eukaryotic Translation Initiation Factor 2 Alpha Kinase 2)(3 transcript variants)

Variant 2 ΔG = -76.30 kcal/mol

```
  1 gaaactgaga accagctctc ccgaagccgc gggtctccgg ccggcggcgg cggcggcggc
 61 ggcggcggcg cagtttctgg agcaaattca gtttgccttc ctggatttgt aaattgtaat
121 gacctcaaaa ctttagcagt tcttccatct gactcaggtt tgcttctctg gcggtcttca
181 gaatcaacat ccacacttcc gtgattatct gcgtgcattt tggacaaagc ttccaaccag
241 gatacgggaa gaagaa
```

Variant 1 ΔG = /

```
1 gatacgggaa gaagaa
```

PRNP (Prion Protein)(5 transcript variants)

Variant 1 ΔG = -23.60 kcal/mol

```
 1 gccagtcgct gacagccgcg gcgccgcgag cttctcctct cctcacgacc gaggcagagc
61 agtcatt
```

SKP1 (S-Phase Kinase Associated Protein 1)(2 transcript variants)

Variant 1 ΔG = -60.90 kcal/mol

```
  1 agccgcgatg tgacgccgcg cgccccgggg tcctcggcgc ctgcgccctc tcctataaag
 61 cagacgccgc gccgcgctgc gacgctgtag tggcttcgtc ttcggttttt ctcttccttc
121 gctaacgcct cccggctctc gtcagcctcc cgccggccgt ctccttaaca ccgaacacc
```

RAB14 (RAB14, Member RAS Oncogene Family)(1 transcript variant)

ΔG = -77.80 kcal/mol

```
  1 atcttggacc agcccgagga agatactgag ggagcacagg agcagtcacc gctgccactg
 61 ctactgccgc tactgctgcc ggcgcgtctg cacctctcgg cctgccagtg tacctgccgg
121 cgcctcggtc gaccgccccc gccccctctc ccgctgcgtc cgcactcctg ttcctggtcc
181 tgacgccccc ctcccgcccg gaaagctgcc cagccaccag caacccccca gtgccacc
```

TFPI (Tissue Factor Pathway Inhibitor)(6 transcript variants)

Variant 1 ΔG = -52.70 kcal/mol

```
  1 agacagcagc gactttaggc tggataatag tcaaattctt acctcgctct ttcactgcta
 61 gtaagatcag attgcgtttc tttcagttac tcttcaatcg ccagtttctt gatctgcttc
121 taaaagaaga agtagagaag ataaatcctg tcttcaatac ctggaaggaa aaacaaaata
181 acctcaactc cgttttgaaa aaaacattcc aagaactttc atcagagatt ttacttag
```

ALCAM (Activated Leukocyte Cell Adhesion Molecule)(4 transcript variants)

Variant 2 ΔG = -206.00 kcal/mol

```
  1 agagcagccc ggagaccgct gccgccgctg ccgctgctac caccgctgcc acctgaggag
 61 acccgccgcc cccccgtcgc cgcctcctgc gagtccttct tagcacctgg cgtttcatgc
121 acattgccac tgccattatt attatcattc caatacaagg aaaataaaag aagataccag
181 cgaaaagaac cgcttacacc tttccgaatt actcaagtgt ctcctggaaa cagagggtcg
241 ttgtccccgg aggagcagcc gaagggcccg tgggctggtg ttgaccggga gggaggagga
301 gttgggggca ttgcgtggtg gaaagttgcg tgcggcagag aaccgaaggt gcagcgccac
361 agcccagggg acggtgtgtc tgggagaaga cgctgcccct gcgtcgggac ccgccagcgc
421 gcgggcaccg cggggcccgg gacgacgccc cctcctgcgg cgtggactcc gtcagtggcc
481 caccaagaag gaggaggaat
```

Variant 1 ΔG = -206.00 kcal/mol

```
  1 agagcagccc ggagaccgct gccgccgctg ccgctgctac caccgctgcc acctgaggag
 61 acccgccgcc cccccgtcgc cgcctcctgc gagtccttct tagcacctgg cgtttcatgc
121 acattgccac tgccattatt attatcattc caatacaagg aaaataaaag aagataccag
181 cgaaaagaac cgcttacacc tttccgaatt actcaagtgt ctcctggaaa cagagggtcg
241 ttgtccccgg aggagcagcc gaagggcccg tgggctggtg ttgaccggga gggaggagga
301 gttgggggca ttgcgtggtg gaaagttgcg tgcggcagag aaccgaaggt gcagcgccac
361 agcccagggg acggtgtgtc tgggagaaga cgctgcccct gcgtcgggac ccgccagcgc
421 gcgggcaccg cggggcccgg gacgacgccc cctcctgcgg cgtggactcc gtcagtggcc
481 caccaagaag gaggaggaat
```

C1GALT1 (Core 1 Synthase, Glycoprotein-N-Acetylgalactosamine 3-Beta-Galactosyltransferase 1)(1 transcript variant)

ΔG = -166.40 kcal/mol

```
  1 aggagacttc gggtggcggc tgcgccatag gcgggccacg gcctggacgc gctgcgggga
```

```
 61 ggggcggagc gagcgggcgg gagcgcgcgc tgggcccgcc ttggccgccg ccgctgtgct
121 gccgctgccg gggaataatc tgggcggcag cgggcggcct cggctagcgg ccacgagcca
181 cttctgcggc tgcccagaga agcaaaggtc accagtccca agtcgtcccc ctctccgccc
241 cccaggaggg gcgagaggga gccgcagctg atgtcagaaa tacactttcg ggaa
```

CLDND1 (Claudin Domain Containing 1)(7 transcript variants)

Variant 1 ΔG = -16.10 kcal/mol

```
  1 agggtgctgt ggtctgagct agagggtgaa gctggcggag caggaggatg ggcgagcagt
 61 ctgaatgcca ga
```

ZNHIT6 (Zinc Finger HIT-Type Containing 6)(2 transcript variants)

ΔG = -53.60 kcal/mol

```
  1 agtcgcgtca agcaggctat tccgggtttg tggctgcttg gctccacacg tgggccgccg
 61 taggtattcc gaccggtaat tcctcctatt ggtgtgcagc agccacattg aaggatagag
121 tggcagcaga ggccaaggat cgtgagttg
```

ZMYM4 (Zinc Finger MYM-Type Containing 4)(4 transcript variants)

Variant 1 ΔG = -176.70 kcal/mol

```
  1 gatttggtga tccctttaag aaaccgcagg cggaggaatt tctctgagag aaaataatcc
 61 tactcacggg gccccttgga ggccattaac cccccgagtc ccggccccca ccccgtcccc
121 gggcaggccc tcccgcccac gcgcggaccc gtgggatctc agaagctgcg gcccggcgcg
181 cggcatccgc cccctcccca ctctcggcgc aaggcccggc cgggtccggg gaagctgccg
241 cgaggcggcc gtgcctgcag tgtgggcggg ggccgggggg ccgagaggta ccgccgccac
301 cgcgcgggga gccgcagcgg ttccgagcgg ggcccaac
```

RAP1A (Ras-related protein Rap-1A)(5 transcript variants)

Variant 3 ΔG = -77.40 kcal/mol

```
  1 caaccacgcc cgcggaccga cccggtcgac tgtaggcctg cgggagaccg tcgggagggg
 61 ccccggccgg ctgtgggaga gaaagaggac attgtgtccc ccacccttca gctccccgag
121 gtgtcatagc acaagcccat ttccctcgcc gtctccccag acacggtgcc ctcgtacctc
181 actgcctgag agaagtagaa gatcgtcagt atttaaacag atcacatc
```

Variant 1 ΔG = -111.20 kcal/mol

```
  1 ggcgccgccg ccgctcccga ggcccctgcc gccgccgctc ccgctgctgt cgccgcgcag
 61 agccggagca ggagccacgg ccgagaggag ggaggaggag gaggaggagg tggaggaggt
121 ggaggaggtg gaggaggcgc cggaccgggg gggatagatt cccagaagtg ggataactgg
181 atcagagggt gattaccctg tgtataagag tatgtgtctc actgcacctt caatggcatt
241 gagtagatcg tcagtattta aacagatcac atc
```

RAB18 (Ras-related protein Rab-18/ RAB18 Small GTPase)(5 transcript variants)

Variant 4 ΔG = -88.70 kcal/mol

```
  1 ggggattctg ggtcgcgcag ccgtggagct acctcaaggc ggaactgggg agtccagggc
 61 ttcggcagcc tgaacacccg gtgtgaaagg aaggtggcag acgtggagcg gcgcgcatgc
121 gcagcagctc actctgctga agggctgaga ggcgcacccg ggcggccagc tgggctcgga
181 gcggaacggg gtcagg
```

Variant 1 ΔG = -24.10 kcal/mol

1 actctgctga agggctgaga ggcgcacccg ggcggccagc tgggctcgga gcggaacggg
61 gtcagg

ARL1 (ADP-ribosylation factor-like protein 1)(2 transcript variants)

Variant 1 ΔG = -45.60 kcal/mol

1 cccgcccccg cctggagtcc gacgtggaag ttgctggctg actgggcttg cgaggaaacc
61 gcctcggagc tgcagccgaa ggccaaggaa tcactgaaga tcggcgaggg aggacagggg
121 gttcatc

CFL2 (cofilin 2)(5 transcript variants)

Variant 2 ΔG = -17.60 kcal/mol
1 agtgccacag agccgaagcc cgagctgccg ccgcagccac agccgagggc act

Variant 1 ΔG = -57.80 kcal/mol

1 acagccgagg gcactatggt aagacgagcg cgctggccgg gaggccgcgg cgaggcgaga
61 aaagcccccc gcacggcccc gggagtgagg ccgccatttt aacctgattt tggcctccaa
121 ggcttctgga gttacagtga atgatgaagt catcaaagtt tttaatgat

FAM13B (Family With Sequence Similarity 13 Member B)(3 transcript variants)

Variant 1 ΔG = -192.60 kcal/mol

1 acgcacgtca cgtccggccc ggcctccgcc cgccagccgc tctcctcccg ctcgccgtta
61 gggaggctct gcacctcagc cgccgcctct gtcgccactc tctccccctg ttccgcgtct
121 gcgtcgccac caccgccggg ggtatccggg gggtcgctta ccgggctggc cccgcgccac
181 agccgtagct ttaacctccc gtcagcggcc gccccaggaa gaggcttctc ggccgcggag
241 ccacttgggg cctcagttct cgccctcttg ccgtcgccgc gggtcctgga gaagcggccg
301 cggccaggga acggggcatc gcggttccaa cgattaactg ctgaagtact gatcgagttc
361 tgcatttctt caatgaggaa ctacaggctg atcttctgcc ataatctcaa acagccataa
421 atgacaaaag aatgcttgct cagtgagggt agctggtgca gaagccattt tttaaactta
481 ggtatttaag tactgaaaga aaagacagct ttgatttctg gctgcaaaaa agat

ASPH (Aspartyl/asparaginyl beta-hydroxylase)(12 transcript variants)

Variant 2 ΔG = -27.10 kcal/mol

1 agtctcaagc tctggtaggc aagtgcatgc agtgtgccta aaacctgcca gcagtacttt
61 tgagtttttt tttttgtttt gttttacttt agcatttatt attcatggat tgaagaaatc
121 aaa

Variant 1 ΔG = -109.40 kcal/mol

1 agtcacaggt tcgtacacgc gaggccgggc gcgcgcagcc ggcaggcggg tgaagtcttc
61 ccaggtgctg caggcggtgc tgaggcacag ggtctgctgc aggagcagcg gcccgaaccc
121 gctccagcgg cccgccgccg ccagcggttc ccgcgtcgcg tgtgtacccc cgcgcactga
181 aggaggtccg ccagccctca ccagccccg cggaccgtgc a

TRAM1 (Translocation Associated Membrane Protein 1)(3 transcript variants)

Variant 3 ΔG = -285.20 kcal/mol

1 gggactgcca gcctcggccg cggtgagcgt cagccccgcg agggccgggg aaggccgagg

```
 61 cccggccccc gggtctgagg agtgccggcc tggagaccac aggccccgag tgacagggga
121 gcccccggta gctccgagat tggggtgagc gagaaaccgg agtcaggcat ctgctttcga
181 cttgaagggc ggtggccgcc gggcaccggc gcagtggagg cgcgccagat tgttttcatt
241 tttgtctttt aaactgatat tattaccagt taacgaactt tttgccctga cgctttaaag
301 aaacaaggtc tgatggataa atcgaggttc tgaacagcgg tgccaaggaa tcgagtaatt
361 ttgtttcttg aagtaggaga ggatgggaat gtcaggagtg tgtaaattct cttcccacgt
421 atctagtatt ttgatgactt atttaacagc tgttgaatgt ggatgataaa ctttgcaagg
481 tgaacgagta acatcctacg tttttcccct cgctccccaa tacacacgct cattcctttg
541 ctttttttccc tttttaattt ttctctggag cctttatgtc tcagtaaata tacaaattat
601 tttgctcctg ccttgttttg tctcctctct cctgcatgta acaagtgaga gtagagattt
661 tggagtattt ttacgagtct gtatttccag agtctaggat agtgcctggg acataggagt
721 gcttaaaaaa agatgagcga cgttaagaat ccttgagaaa acctaatgga atgatattac
781 cagtgaccaa aagataacgg caaaagcttc tatcattttt gttactcttc agtacaatgt
841 caccctccca gcaacagaag aacaagctac tgaatcagtg tccctttatt actatggcat
901 caaagatttg gctactgttt tcttctac
```

Variant 1 ΔG = -106.10 kcal/mol

```
  1 atgctcgccc ccgctgcggg ctagctgttg tgtttttttt tttcccccgg gcggcccggc
 61 ggctgcgtac tggctgtggg atgggaagtg aagccccagc gagcggctgc agcggggccg
121 tgaggagcag ccagcgggag gcggcggcga gtcggtgagc agctgggaag agcagaaccg
181 gggcggagca cctgcaggcg cgggcggcgg ccccacc
```

FAM3C (Family With Sequence Similarity 3 Member C)(2 transcript variants)

Variant 1 ΔG = -78.00 kcal/mol

```
  1 acttgccggg gtgaggggca gccggaggag tccgagagga agcggaggcg cgagctggag
 61 gcggcggctc ccgtcggcct ccggcaggac tgagcgctgg gaggccggaa ggcgggcgcg
121 cgcggcggag aggcgggcgg gaggccggag catattaatg aaaagtgcca taaactgaaa
181 aaccaaac
```

mRNA-changed; RPF-unchanged (padj < 0.05 & resLA_padj < 0.1)

PTP4A1 (Protein Tyrosine Phosphatase 4A1)(1 transcript variant)

ΔG = -406.30 kcal/mol

```
   1 aaagatgacc gtggttaagg cagtagaaag caggctcgac actgagacgc ggttccagcc
  61 ctagaaggat tgcattttac gattcaggca aacttcgagt ctcctcatgt gacatgtgtg
 121 cactcctgga ccgttttaat tatgagaaac gtggtttgca gcatgattcc ttccagtcga
 181 taaatcggaa tctctctcgc tcccacccct tcttaacttc aggcttcctg catcccggag
 241 cactcccggc agccccttcc ctccccccgcc ccggggatgc tccgactcgg cgcttagcca
 301 ttcatcaacc ggttcacacc ggcggcggcc gccgcggagt gacgtccgga gggggcgggc
 361 ctccgccccc gcctgtcggc tcctggcccg cggttccagg ccgcgattgg tggctggagg
 421 gttgcacgtc gcgccggcta taaaggggag ggcttgtgac gcaagggcgc ctcggcgcgt
 481 gtattggctc cttcggctgc gggccggctc ggctacgcgc tctgctccga gccgctcact
 541 gcatggtaga gtctggtgcc cccgccgccg cctgcatcgc cgccaccgcc gctccgccac
 601 gaccaccgcc gcctcctgcc ctgcagccac cgccaccgcc tgtgtcgccg ccgcctcggg
 661 accggctgta tgattaggcc acaatcttca atgagtaaac atattcctca attctgtggt
 721 gttcttggtc acacatttat ggagtttctg aagggcagtg gagattactg ccaggcacag
 781 cacgacctct atgcagacaa gtgaactgta gaaactgatt actgctccac caagaagccc
 841 ccataagagt ggttatcctg gacacagaag tgttgaattg aaatccacag agcattttac
 901 aagagttctg acctggatgg ggtaaacctc agtgcacttc ttttctgttg gcctcagtat
 961 tactggattg aagaattgct gcttcttgtt aggaggttca tttcacttat cattacttac
1021 aacttcatac tcaaagcact gagaatttca agtggagtat attgaagtag acttcagttt
1081 ctttgcatca tttctgtatt caattttttt aattatttca taaccctatt gagtgttttt
1141 taactaaatt aac
```

RALGPS2 (Ral GEF With PH Domain And SH3 Binding Motif 2/ Ras-Specific Guanine Nucleotide-Releasing Factor RalGPS2)(10 transcript variants)

Variant 10   ΔG = -117.00 kcal/mol

```
   1 actctcctcc cccgagcggc agcggcggcg gcggcggcgg ctgctgcggg cgctgaatga
  61 gagacggtga ctgttcgggt cgacgagtgc tactctaggc ggcggcggcc gtggcggtga
 121 agcgtgaggc cggcatcgtc tttccgtcct ctgaggcgac ggccgcggct gcacaggaat
 181 aatgtatttg tggccttgga catgaggcag tcagtcctct gttgctgtta acataaggtc
 241 agggactgat gaggaaagc
```

UBE2W (Ubiquitin Conjugating Enzyme E2 W)(6 transcript variants)

Variant 2   ΔG = -11.40 kcal/mol

```
   1 gcgtctctgg cctcgccggt cttgggggga tggttccatc
```

Variant 1   ΔG = -11.40 kcal/mol

```
   1 gcgtctctgg cctcgccggt cttgggggga tggttccatc
```

ATP2B4 (ATPase Plasma Membrane Ca2+ Transporting 4)(4 transcript variants)

Variant 2   ΔG = -313.80 kcal/mol

```
   1 gagttagcta gagctgttgc catgacaagc attttcttaa gaaagctctg ctgttgagat
  61 cgtccaaagc tgggggctgg gagggggggc acgaacttcg tggggcacca tccctccgaa
```

```
121 gagagcaaga tcagagacac ccgcccagct ctcctgcacc ctttttcttt caggaacctg
181 agaggtcctt cccattggag gggcaggggg aggagaccca gcccactccc cggccttagc
241 cagaaaggca agagagcaaa gccagcgtct ctagcttggt tgccacactg gggagactgg
301 tgggtggtca ccccacccta ccctcatcca tggaaacccg gagggagagt cccgcctgaa
361 gaaacctgga tctttgctag aaagagagag atccagttgt catcggtatc caggaagctc
421 tcctcttcct cctcctgacg tctactacta cagttgctgg ttgttgctaa ggttgctgcc
481 atggtaacat gcacatcctg tttacacctt catctgggca agttggtcta agctaggaac
541 ctacctaccc tggacaacta ctatcatcac cacctgggga caccaatcat cgtgacacgg
601 agtccacctt ccactcagtt cccccatcct cttcctcctc tcgctgccag acttcatacg
661 gaagaaagga tctagacttc ggacggctac tcgggagctt attgcacaag atatattcaa
721 tctattccct cactgggccc ccagagaagc aagaagtagg aagaagttga gacagggagg
781 caggagacac tggtcagttg aagggaaacg ctacatcttc tctggttgag ggcttggta
841 acagcaggca aa
```

Variant 1 ΔG = -333.50 kcal/mol

```
  1 cttcccgcac tgcttgggga gagctggtga gcaagagtct ggcccgagtt agctagagct
 61 gttgccatga caagcatttt cttaagaaag ctctgctgtt gagatcgtcc aaagctgggg
121 gctgggaggg ggggcacgaa cttcgtgggg caccatccct ccgaagagag caagatcaga
181 gacacccgcc cagctctcct gcaccctttt tctttcagga acctgagagg tccttcccat
241 tggaggggca gggggaggag acccagccca ctccccggcc ttagccagaa aggcaagaga
301 gcaaagccag cgtctctagc ttggttgcca cactggggag actggtgggt ggtcacccca
361 ccctaccctc atccatggaa acccggaggg agagtcccgc ctgaagaaac ctggatcttt
421 gctagaaaga gagagatcca gttgtcatcg gtatccagga agctctcctc ttcctcctcc
481 tgacgtctac tactacagtt gctggttgtt gctaaggttg ctgccatggt aacatgcaca
541 tcctgtttac accttcatct gggcaagttg gtctaagcta ggaacctacc taccctggac
601 aactactatc atcaccacct ggggacacca atcatcgtga cacggagtcc accttccact
661 cagttccccc atcctcttcc tcctctcgct gccagacttc atacggaaga aaggatctag
721 acttcggacg gctactcggg agcttattgc acaagatata ttcaatctat tccctcactg
781 ggcccccaga gaagcaagaa gtaggaagaa gttgagacag ggaggcagga gacactggtc
841 agttgaaggg aaacgctaca tcttctctgg ttgaggggct tggtaacagc aggcaaa
```

ITGA4 (Integrin Subunit Alpha 4)(2 transcript variants)

Variant 2 ΔG = -323.60 kcal/mol

```
  1 ataacgtctt tgtcactaaa atgttcccca ggggccttcg gcgagtcttt ttgtttggtt
 61 ttttgttttt aatctgtggc tcttgataat ttatctagtg gttgcctaca cctgaaaaac
121 aagacacagt gtttaactat caacgaaaga actggacggc tccccgccgc agtcccactc
181 cccgagtttg tggctggcat ttgggccacg ccgggctggg cggtcacagc gaggggcgcg
241 cagtttgggg tcacacagct ccgcttctag gccccaacca ccgttaaaag gggaagcccg
301 tgccccatca ggtccgctct tgctgagccc agagccatcc cgcgctctgc gggctgggag
361 gcccgggcca ggacgcgagt cctgcgcagc cgaggttccc cagcgccccc tgcagccgcg
421 cgtaggcaga gacggagccc ggccctgcgc ctccgcacca cgcccgggac cccacccagc
481 ggcccgtacc cggagaagca gcgcgagcac ccgaagctcc cggctggcgg cagaaaccgg
541 gagtggggcc gggcgagtgc gcggcatccc aggccggccc gaacgctccg cccgcggtgg
601 gccgacttcc cctcctcttc cctctctcct tcctttagcc cgctggcgcc ggacacgctg
661 cgcctcatct cttggggcgt tcttccccgt tggccaaccg tcgcatcccg tgcaactttg
721 gggtagtggc cgtttagtgt tgaatgttcc ccaccgagag cgc
```

Variant 1 ΔG = -106.10 kcal/mol

```
  1 gaagcagcgc gagcacccga agctcccggc tggcggcaga aaccgggagt ggggccgggc
 61 gagtgcgcgg catcccaggc cggcccgaac gctccgcccg cggtgggccg acttcccctc
121 ctcttccctc tctccttcct ttagcccgct ggcgccggac acgctgcgcc tcatctcttg
181 gggcgttctt ccccgttggc caaccgtcgc atcccgtgca actttggggt agtggccgtt
241 tagtgttgaa tgttccccac cgagagcgc
```

NAV1 (Neuron Navigator 1/ Pore Membrane And/Or Filament Interacting Like Protein 3)(2 transcript variants)

Variant 1 ΔG = -62.80 kcal/mol

```
  1 ggagccgcgg ggcttccatc cttcctttga ctgattttta aattttaatt tgtattttcc
 61 ccgccgcccc gccccttttc ctccgacccc gccctatcgc tccccggctt ccctgctctt
121 tcctttttcc cggcttcctt cctcgcgttt ctttcccctg cgccctcggc ttgcctctct
181 ccctcctccc tcgctctctc ccccttctct cccctttcttc ctcggtttct tccgtcctct
241 ctctccccct cctcctcccc cgcctcctcc tcctgcgctc ccgccccctg ccccctcccc
301 ccgtgcctgc agacgcgcgg atcgtccatg cgctcctcgc gggcaga
```

LRRC58 (Leucine Rich Repeat Containing 58)(1 transcript variant)

ΔG = -57.20 kcal/mol

```
  1 acttccgact cagccctcgg ttcaggttcc ggggcgccgc agagctcccg gcctctggac
 61 cgcgcgcggc gctctgggga atccggcgcc acgcgccgtg cggtggccag g
```

SIRT1 (NAD-Dependent Protein Deacetylase Sirtuin-1)(3 transcript variants)

Variant 1 ΔG = -20.70 kcal/mol

```
  1 gccagtgccg cgcgtcgagc gggagcagag gaggcgaggg aggagggcca gagaggcagt
 61 tggaag
```

SF3A1 (Splicing Factor 3a Subunit 1)(1 transcript variant)

ΔG = -37.90 kcal/mol

```
  1 gccatcttgc gagctcgtcg tactgaccga gcggggaggc tgtcttgagg cggcaccgct
 61 caccgacacc gaggcggact ggcagccctg agcgtcgcag tc
```

NCOA7 (Nuclear Receptor Coactivator 7)(6 transcript variants)

Variant 4 ΔG = -133.60 kcal/mol

```
  1 gcaattacca aaggagtcaa gacaccttac agcccagcct taattcagaa accctcataa
 61 agtagatgca gagggcagta agatataact caactttgaa aatgtcagcc gttatagttg
121 aagaaatctg acccaagaga cttcgctccg ctgcaagatg gaaggaagct taagtaagac
181 ataaatttgt aatgaacttg ctcacaacat ccgccgccac tgtgacttgc agtcatcatc
241 cattaccaca aaattagttg caggatggct actcgtatcc ctccacacat gatcatcagt
301 atttgcctcc tgtgtcccaa ccggcctgag tcaaattccc actctgtcgc ccaggctgga
361 gtgcagtgat gtaatctcgg ctcactgcaa cctccacttc ccaggttcaa gtgattctcc
421 tgcctcagcc tccagagtag ctgggattac agggttacga ctcactgatt aaaaagaggg
481 actttttcaa atactttgca cttttgattg tgtatt
```

Variant 1 ΔG = -44.90 kcal/mol

```
  1 gagaggatgc gctccccggc gcccagcagc agaggccacc gctcccagaa atgcatgcga
 61 ccgatcccct tctcccggac cccaggagcc ggcgcccccg ccctgtaggg ttacgactca
121 ctgattaaaa agagggactt tttcaaatac tttgcacttt tgattgtgta tt
```

CPEB2 (Cytoplasmic Polyadenylation Element Binding Protein 2)(6 (A-F) transcript variants)

Variant D ΔG = -95.70 kcal/mol

```
  1 gcgacggcgg cggcggcggc gatcgccgcg aggggtggtg gggccgaagt cggtgccccc
 61 tggctcagtc acggtgtccc tctctcactg actccccctc cttccaccac ggccgcgcaa
121 ccccagcgcc ggcggcttcc taggtggggc aggggacgag gagcgtctcc tcccgctgcc
181 ggcggcctga taa
```

Variant A ΔG = -95.70 kcal/mol

```
  1 gcgacggcgg cggcggcggc gatcgccgcg aggggtggtg gggccgaagt cggtgccccc
 61 tggctcagtc acggtgtccc tctctcactg actccccctc cttccaccac ggccgcgcaa
121 ccccagcgcc ggcggcttcc taggtggggc aggggacgag gagcgtctcc tcccgctgcc
181 ggcggcctga taa
```

FBXO30 (F-Box Protein 30)(2 transcript variants)

Variant 2 ΔG = -42.20 kcal/mol

```
  1 gcgcgaggag ggtggctgcc tctggagcag gccgggagaa gagaaaaggc ggcggcccgg
 61 ctggggaaga ggggcggagg aggcggttcg gacggctgtg tgggagctgg actggccatt
```

Variant 1 ΔG = -96.60 kcal/mol

```
  1 gcgcgaggag ggtggctgcc tctggagcag gccgggagaa gagaaaaggc ggcggcccgg
 61 ctggggaaga ggggcggagg aggcggttcg gacggctgtg tggggtacgg agcgtcgccg
121 ggacagagcg gagcagctgg gcacggcgtc cagggcagct agggcctggg ccgcggttcg
181 gccgcgccgg ccgagctgga ctggccatt
```

CDH6 (Cadherin 6)(2 transcript variants)

Variant 2 ΔG = -120.50 kcal/mol

```
  1 cccacttcat tcacttgcaa atcagtgtgt gcccacaaga gccagctctc ccgagcccgt
 61 aaccttcgca tcccaagagc tgcagtttca gccgcgacag caagaacggc agagccggcg
121 accgcggcgg cggcggcggc ggaggcagga gcagcctggg cgggtcgcag ggtctccgcg
181 ggcgcaggaa ggcgagcaga gatatcctct gagagccaag caaagaacat taaggaagga
241 aggaggaatg aggctggata cggtgcagtg aaaaaggcac ttccaagagt ggggcactca
301 ctacgcacag actcgacggt gccatcagc
```

Variant 1 ΔG = -120.50 kcal/mol

```
  1 cccacttcat tcacttgcaa atcagtgtgt gcccacaaga gccagctctc ccgagcccgt
 61 aaccttcgca tcccaagagc tgcagtttca gccgcgacag caagaacggc agagccggcg
121 accgcggcgg cggcggcggc ggaggcagga gcagcctggg cgggtcgcag ggtctccgcg
181 ggcgcaggaa ggcgagcaga gatatcctct gagagccaag caaagaacat taaggaagga
241 aggaggaatg aggctggata cggtgcagtg aaaaaggcac ttccaagagt ggggcactca
301 ctacgcacag actcgacggt gccatcagc
```

HNRNPR (Heterogeneous Nuclear Ribonucleoprotein R)(7 transcript variants)

Variant 7 ΔG = -60.30 kcal/mol

```
  1 gccatttcta gtcgttttca aagcgcctcg cgctgattct cacgggcccg gctgccggcc
 61 cccgctctgc cctggattgg tagcttatgt cgatcttgat gaaagagcaa ttgatgctct
121 cagggaattt aatgaagaag gagctctgtc tgtactacag cagttcaagg aaagtgactt
181 atcacatgtt cagaacaaaa gtgcattttt atgtggagtt
```

Variant 1 ΔG = -18.50 kcal/mol

```
  1 gccatttcta gtcgttttca aagcgcctcg cgctgattct cacgggcccg gctgccggcc
 61 cccgctctgc cctgcataat aaa
```

RNF2 (RING-Type E3 Ubiquitin Transferase RING2)(1 transcript variant)

ΔG = -46.60 kcal/mol

```
  1 atattgtgcg gcggcgccgg cgtccgcggc agctgatacc agagtcttgc tccggccgcg
 61 gccagcggag ccctgggctg gggcaggagc cgca
```

BRD1 (Bromodomain Containing 1)(7 transcript variants)

Variant 1 ΔG = -235.40 kcal/mol

```
  1 gcattgtttg cttcgctggg gagcgagcga gcgacccggc cctggagtcc gagccagccc
 61 cgcgtcccgc gcccgccgcc cgccgcccgc tcccgcgccc gcgcccgcgc ccgccggccg
121 ggcccccga gccgggcctc cagctgggcc ccgagcccga gcccgcgccg cgcgagccgc
181 tgcccgagag tccgcggccg ccgctgccgg gcccggagcc gccgcggccg agaggggccc
241 gcgcccagga gcggcggcgg ggcccggcct gcgcggccgt tggcggagga gccgcgggcg
301 ccattagcac cgcctcggcc gcgccggccc ccgcgcccgc ccgcttgccg ggctcccgcg
361 gccgcggcgc cccgaaggta atcattacca a
```

FARP1 (FERM, ARH/RhoGEF And Pleckstrin Domain Protein 1)(3 transcript variants)

Variant 1 ΔG = -244.30 kcal/mol

```
  1 gcacttcccg ctcgccggcc tcagaggcgg cgggtccggc gcgggcgcag cggtgcgggc
 61 gctcggctgg ggcgcggggc ggggacgcgg ccgctgcccg ctttgcgccg ctcctccctg
121 cgcgagtagc gctggccccg gcgtcgaggc ggccatggcg acccggagcc cgctccccac
181 ccaccccgcc tgctccgccc tcccctccgc cccgcgccac ctttgatggc tcggacctca
241 gccggccacc gccagccctg ctcgcgcgcc cgcgccgccg ccgcccgcgg gtattaatag
301 ccggcgccgc cgcgccctcg gccgccgggg gcttgggagc cgccgatccc ggagcccgag
361 ccgggagagg gagccgccgc agccgccggc gctgtggaga tattctctaa gccgctttca
421 tc
```

INPP5F (Inositol Polyphosphate-5-Phosphatase F)(3 transcript variants)

Variant 1 ΔG = -118.50 kcal/mol

```
  1 gagaggcctc tacggccgcc gctgccgccg ccgctgccgg ggcgcgttct cctcctaccg
 61 gtcgggtgcc ccggggcgtt ccctctgccg ctgcttctcg gcgcggttcc tacccggccg
121 ctccccgagg cgcgggctct ggcggcctcg accgactagg acgccccgtg cgccgcccgc
181 gggccgccgc ctccctgggc gcgcggggcc agc
```

USP40 (Ubiquitin Specific Peptidase 40)(2 transcript variants)

Variant 2 ΔG = 1.80 kcal/mol

```
  1 ggtagtattt agtttcaca
```

Variant 1 ΔG = -50.20 kcal/mol

```
  1 actttctgga gacccgccct cgccaacatg gcggcgccca gttgggcgg gttcgttcgc
 61 ttcgcgtttt ggccagggcg ggggtctggg ctttaggcag gtagtattta gtttcaca
```

PPP1R15B (Protein Phosphatase 1 Regulatory Subunit 15B)(1 transcript variant)

ΔG = -156.80 kcal/mol

```
  1 attttgggct tcgcttccac cgcaccagcc ggcctaccca gtccttccgg tatcgcgttg
 61 ctcaggggct tttcaaccct ctgtcagtcg gaaaaccatc gccgaggccg tggggggact
121 cctatccatg gtgttgaagc gtcgagccga ctagggaacc tccttccccg ccaggatgga
181 agtcgcatca gtcgccgcct attgcgcggg ctgttcttcc ctgtgttctg ccgcccgctg
241 ccgcattcgc tgccctctgt ggcttttctg ctggctcgaa gatcggcctg gagcagcgac
301 gccaccgctg ggcaaggccg agactctgta ggcttcctcc gaatcccgtc gacctccagc
361 cgctgagcgc cgcggcccta cctgagagac tgtcaagaaa aaggag
```

KRAS (KRAS Proto-Oncogene, GTPase)(4 transcript variants (a-d))

Variant c   ΔG = -93.50 kcal/mol

```
  1 ctaggcggcg gccgcggcgg cggaggcagc agcggcggcg gcagtggcgg cggcgaaggt
 61 ggcggcggct cggccagtac tcccggcccc cgccatttcg gactgggagc gagcgcggcg
121 caggcactga aggcggcggc ggggccagag gctcagcggc tcccaggcct gctgaaa
```

Variant a   ΔG = -96.50 kcal/mol

```
  1 ctaggcggcg gccgcggcgg cggaggcagc agcggcggcg gcagtggcgg cggcgaaggt
 61 ggcggcggct cggccagtac tcccggcccc cgccatttcg gactgggagc gagcgcggcg
121 caggcactga aggcggcggc ggggccagag gctcagcggc tcccaggtgc gggagagagg
181 cctgctgaaa
```

EXOC5 (Exocyst complex component 5)(1 transcript variant)

ΔG = -125.40 kcal/mol

```
  1 agttccactt ccggcgtatg aggcggtgac aatgggagca gcgcgggcgg cgccgggagg
 61 cagctgacaa gcgtttgcgg cttcgcttca tggccgctct cccgcccctc ctgggatctg
121 tggggagctg gggagcccgc agcggcccgg agccggagct ggcgagccga gcggagacct
181 gtgcgccgcg cctctgaggc gcagcatgtg aagcggagac ggcatccagt gggggcgag
241 cctctcagcc ggccggg
```

MAP1A (Microtubule Associated Protein 1A)(1 transcript variant)

ΔG = -155.80 kcal/mol

```
  1 actcccaccc taagtgctgc agactcttcc ctgaagctgc cggctgaggc cggagctgcc
 61 gcctccatga gaggcttcct cctacacccc agggccagag gaccctttgc caccagagtg
121 agatcctaga gaccatcatc ctggtaaatc ccagtgcaga cagcatcagc tctgaggttc
181 atcatcttct tagcagctca tcagcttata aactactaat cttgagtggg caaagtttag
241 agcctggggg agacctcatc ctacagagtg gcacctactc atatgaaaac tttgcccagg
301 tccttcacaa ccccgagatt tcccaattgc tcagcaatag agaccctggg atacaggcct
361 tccttaccgt gtcctgctta ggggaaggtg attggagcca cctgggatta tccagttccc
421 aagagaccct gcacctccgg ctaaaccctg agcccactct gcccacc
```

PRKACA (cAMP-dependent protein kinase catalytic subunit alpha)(3 transcript variants)

Variant 3   ΔG = -126.40 kcal/mol

```
  1 aaataaaaaa taataatagc tgacatttat ctggcactat catcacacgg ggccactgtt
 61 caactcccctt tgtcaagatg aactcattca ttcctcaaaa ccacccccact ttacagatga
121 ggaaaactga ggcctagaga ggtgagatca gttacccaaa gtcacagagt tcatcagaga
181 ccaggctggg ctctgaactg gatcttgacc ccaagtcctg gcccttagcc actttgctgg
241 gctgtgtctc agcagagacg agaatgactg ttttcaagcc tgtttcctgg gttgttaagt
301 ggagacaatg gatacaaggc aggttgccga gtgagtcaga tgagacagtg tagacagcag
361 ccggcacagg gtctgccggg gaagtttcaa cattagccag gcggtg
```

Variant 1   ΔG = -124.20 kcal/mol

```
  1 acagacagcg gcagagatct tgggctgagg ttcccgggcg ggcgggcgcg gagagacgcg
```

```
 61 ggaagcaggg gctgggcggg ggtcgcggcg ccgcagctag cgcagccagc ccgagggccg
121 ccgccgccgc cgcccagcgc gctccggggc cgccggccgc agccagcacc cgccgcgccg
181 cagctccggg accggccccg gccgccgccg ccgcg
```

AFF3 (AF4/FMR2 Family Member 3)(2 transcript variants)

Variant 1 ΔG = -88.60 kcal/mol

```
  1 gccattcaga aagctcgcca gcgagaagag agctccacgc tcagcctcag aagaaagacg
 61 ttcaacgact tactggggag agaaagaaaa ggaacgggag ctgagagctg atcacctgcc
121 tttccagacc ggagacgggc ggcaagtttg aagtcgacac ccagaggcca gtttgtcccc
181 cttcccgcgc gcgggcggcc cgagcctcgg cggcggcggt agcggcggcg gcgacgctga
241 cacctcccac c
```

IP6K1 (Inositol Hexakisphosphate Kinase 1)(3 transcript variants)

Variant 1 ΔG = -148.00 kcal/mol

```
  1 agccgccatc ttgttgttga tccgtaccca gtgggcagcg ccgggagctg gaccaagcgg
 61 ccggtgagag gccgctgtag cggtgctcag ccacctgtgc tgcctgccag ggggcgggcc
121 gaaacctgga ggcccggggg gcccagctcc cgtagggagc cgtgggcgct cggtgcccgg
181 gccgggcagg acagaataat aagctgaata gaatctgacc attggctttc acctggccag
241 gaccttctat gtagctctcc ttttgtggcc catgtgctgc atcctctgcc ctcagtgtgc
301 aactggcccc caacgca
```

TES (Testin LIM Domain Protein)(2 transcript variants)

Variant 1 ΔG = -76.00 kcal/mol

```
  1 gctgcggcgg actgggcggc ggaagttcga cggcgccggg cgagtggctg ttgagcggcg
 61 ccgcgggagt tccgcaggtt tcccgtgttc gcagcggagc cggaggccag ctgaacccgg
121 ccgtgggatc ccggatagga ggaggagggg acccatagga cgcgttaac
```

TMEM200A (Transmembrane Protein 200A)(4 transcript variants)

Variant 2 ΔG = -131.00 kcal/mol

```
  1 cttgcacccc ttgccacccg ccccctcgcc tgactcatcc gcccgcggtg gccgcccgag
 61 ccctgggatg gggagggaga ccgcggctgc ccgcggcggc cgagattccc gctgacgccc
121 ccgaccctgc cgccttcttc gtccgcctcc agaggcgccc gacgtcccga cagctcctgg
181 agtgagacca ggactgagaa cagggagagg cgacccgacc cccagggccc ggtgctcagg
241 acagcacaca gagccgctga aaacgactga agagagcaag ggatttcctg ggacatctgg
301 ctctggagag taaaaggcca agct
```

Variant 1 ΔG = -110.10 kcal/mol

```
  1 cttgcacccc ttgccacccg ccccctcgcc tgactcatcc gcccgcggtg gccgcccgag
 61 ccctgggatg gggagggaga ccgcggctgc ccgcggcggc cgagattccc gctgacgccc
121 ccgaccctgc cgccttcttc gtccgcctcc agaggcgccc gacgtcccga cagctcctgg
181 agtgagacca ggactgagaa cagggagagg cgacccgacc cccagggccc ggtgctcagg
241 acagagtaaa aggccaagct
```

SPEN (Spen Family Transcriptional Repressor/ Msx2-interacting protein)(1 transcript variant)

ΔG = -195.70 kcal/mol

```
  1 agtgggaagc gtccggctgc cacagcgcca gctccgtcgt agtcgctgcc gcccgtgtcc
 61 cgctcgcccc tcctcccgct cccccgcccg cccctgcccg ggcgcatgcg ctgccggagc
121 gcgagggtcg gcttcgggtg tgtggtggcg gcagagctga gctgcgaggc ccgagagtca
181 gaacctgggg gagagggatg gtctctgcac gggggggagc cggaggagcc gccgccgctg
241 ccgacgccac cgccgcagcc gccgccgccg ccgccccggc acccgcctcc cggcgctgac
301 ggtctcgtac gaagccggcg agggggagcc agcagcggcg gtcgccggca cgccgcccag
361 c
```

MARCHF7 (membrane associated ring-CH-type finger 7/ Axotrophin)(4 transcript variants)

Variant 2 ΔG = -37.90 kcal/mol

```
  1 ggttctgcgc cggatccggg agaggggcgg gcgccattgt gcttcgctgc cgactgcatt
 61 tcctcagtca cgggcctaga actccaagga gaaaggcggc gaaaaatctt taaga
```

Variant 1 ΔG = -70.40 kcal/mol

```
  1 ggttctgcgc cggatccggg agaggggcgg gcgccattgt gcttcgctgc cgactgcatt
 61 tcctcagtca cgggcctaga actccaagga gaaaggcggc ggaattcatg atttgttcct
121 gtagctgaaa catacattga atgctttctg ccctggcatg aacttacatg gtcagagctg
181 gtttttcctg ccctcaaata tatttctctg actctaccgg ttagactaga aaaatcttta
241 aga
```

PAK2 (P21 (RAC1) Activated Kinase 2/ Serine/threonine-protein kinase PAK 2)(1 transcript variant)

ΔG = -147.10 kcal/mol

```
  1 gtcttcctcc cccagggttg tggccacgcg cagcggcggc ggttgttccg cttcccctcc
 61 ggcccgggcc gtcgccattg ccgaaggctc cctcccctcc cctccctggc gtgcgcagga
121 ctccgccgcc gctgggccta gcggtagcag cggctgctcc agcgcggcgt ctcttcccgc
181 cccgcttccc cttccctccc ctcccctccc cgcaccgcgc gctagcccgg ggcggctccg
241 cagcccgccg ggagctctga ccgaggcgcc tcgctggggc ggggaccttg ccttgcccgg
301 ggccatttca taattctgaa tc
```

PPIP5K2 (Diphosphoinositol Pentakisphosphate Kinase 2/ Inositol hexakisphosphate and diphosphoinositol-pentakisphosphate kinase 2)(12 transcript variants)

Variant 1 ΔG = -112.90 kcal/mol

```
  1 gtagtagcct gaggttccct tatgtggccc tatagctgtt actgaaggaa gtagcctacg
 61 tccacgccta caactgaagt ctcttgacaa acacctcacc cctgcctccg ggatgaaagg
121 gggtaaccta gacctgaatg ggcttgacca tctcacaact gctcgcgtga cgaccgcatt
181 cgtggcaggt aagaagattg ctgtatcaac tcaagaaagc agtaacttca ctgtctttgt
241 attttgaatt gcaacaacaa ctttgatatc aacaatgaag caatgatatc taagaacaaa
301 agagtatttg ccaacagtca tcataatatc aagtgattgt ataagcagaa acaagctgtc
361 acagacctgt gcgtcagcta atatatggag aatgctttct tctgatacta tttacttaga
421 ggcagtttta atataaatca tttcaattat atctacatca aataaaataa aa
```

HSD17B4 (Hydroxysteroid 17-Beta Dehydrogenase 4/ Peroxvariantmal multifunctional enzyme type 2)(5 transcript variants)

Variant 3 ΔG = -17.70 kcal/mol

```
  1 attccccgcc tcctcctgtc ccgcagtcgg cgtccagcgg ctctgcttgt tcgtgtgtgt
```

```
 61 gtcgttgcag gccttattc
```

Variant 1 ΔG = -117.40 kcal/mol

```
  1 attccccgcc tcctcctgtc ccgcagtcgg cgtccagcgg ctctgcttgt tcgtgtgtgt
 61 gtcgttgcag gccttattca tgggctcacc gctgaggttc gacgggcggg tggtactggt
121 caccggcgcg gggcaggtg agcatgcgaa ggttggaggc cgcgcccctt gctgaggcgc
181 agctggctgc tcttttcggg ccggcatacg cgcgcagccg cagctgaggt caccccgctg
241 aggtggtggg gaggggа
```

STC2 (Stanniocalcin 2)(1 transcript variant)

ΔG = -514.30 kcal/mol

```
   1 tttctccttc cctccacggg ccgggtgaga aagtagccgg gggctatccc gacccggcgg
  61 ttcttgggga gggggccgaa caagaaaagg gaggagatgg agataacttc cccggattta
 121 gctttttttgt ctttgttttt gttctcacca cttccatcgg atgactggag agtaaaaggg
 181 aacccggagc ggggtggcga gcagcgcttt gagaaaatgc aggagtgtgt ttggagacgc
 241 gtaaagttgc ctttcaagct ctggcctccg ggcacgcgat gctccgcggc gggctgactc
 301 agggctgcct tgggcctccc tgccaccctc ctggaaatga tgcaagtcct gactgtcacc
 361 tggatccctg cagcccagcc tggaatgcgt ctggattagg ggaaagacga gaaacgacac
 421 tccaggtgtt gcacggccca ccaaagcggg aagatagggc agttgctcag accaaatact
 481 gtatctagtg cttctgctcc tatcttcaat cgtggggttc tttttaatgc aaagtgtcac
 541 aaggccagga attcccatgt gtgctcagtt ggcccacagc atcattgtgc ctaggaaact
 601 gcttcaattt atcaagtcct ctgggctggg aatctcactg aattccaaac ggcggaaaga
 661 ggaaactttc ccaacccgat gtgggtgtga cgcgagccag gggccccagg gacactgtcc
 721 cagagcacac cgtccccctt taacagcaac tggagcttgg attcgctctt atattgtaca
 781 gtcctttcga ccattgccct ggagcacccg cacacgcgca cgcatctccg gccgcgctca
 841 cacacactca tacacacgca cgcaaacgcg tggccgccgc caggtcggca actttgtccg
 901 gcgctcccag cggcgctcgg cttcctcctg tagtagttga gcgcaggccc cgcctcccgg
 961 ccgtgttgtc aaaagggccg gggtctcgga ttggtccagc cgccgggaca acacctgctc
1021 gactccttca ttcaagtgac accagagctt ccagggatat ttgaggcacc atccctgcca
1081 ttgccgggca ctcgcggcgc tgctaacggc ctggtcacat gctctccgga gagctacggg
1141 agggcgctgg gtaacctcta tccgagccgc ggccgcgagg aggagggaaa aggcgagcaa
1201 aaaggaagag tgggaggagg aggggaagcg gcgaaggagg aagaggagga ggaggaagag
1261 gggagcacaa aggatccagg tctcccgacg ggaggttaat accaagaacc
```

PSD3 (Pleckstrin And Sec7 Domain Containing 3)(3 transcript variants)

Variant 1 ΔG = -67.10 kcal/mol

```
  1 ggcagtcccg ggagctcaac aaagagcacg cggcgctggc cgccggcact cgcgccctga
 61 ggctgcggcc ccggagcgcc cggcggcggt ttcggcgcgc ggccgggctg gcgatggaag
```

RAB34 (Ras-Related Protein Rab-34/ RAB34, Member RAS Oncogene Family)(10 transcript variants)

Variant 1 ΔG = -315.20 kcal/mol

```
  1 acagcgttgg cggagcccga gcggagtggg actcgaggcc ctgtagccgg actgggcccc
 61 tcgccccctc ctccagcggc ctgcgggcct tggcagcgcc gcccctgccc acactgaccc
121 cgtgtggcgg ggccgctccg cgcccccggg ccgctctgta atggtagggc agcctcagcc
181 ccgagatgac gtcgggtctc cgcggccccg ggttattgta ggaaccatcc ggccccgcgt
241 aatcgtagga actatccggc cccgcgtaat cgtagggtct gcgcgggccc ggcccccacc
301 agacgggact ccccgccccc aattggcggc cgaagagtct cctcgcccca gagtcatctt
361 cgggacgccc agggcccggg tgattttggg ctcgccgcgg ccccgggtga ttgtttcatc
421 tccgtggccc gcggtggtcg tagcgtctcc gagaccgcgg actcccgtag ggtccccgtg
```

```
481 gccccgagtt gtagtcggga caccccggcc gcgggtgatc gtcgggtctc cacgcgcccg
541 ggtcgctgac gcggatccgg cctcggcgcc ttctcagggc gccctgcaag gccgcaggca
601 gg
```

DCAF7 (DDB1 And CUL4 Associated Factor 7/ Seven-WD-Repeat Protein Of The AN11 Family-1)(2 transcript variants)

Variant 1 ΔG = -66.80 kcal/mol

```
  1 gtcgtccgtt cccaagctgg tttgaaacta ggggtcgggc tcggccgtcg tcgttgtttg
 61 tcgccgcatc cccgcttccg ggttaggccg ttcctgcccg cccccctcctc tcctcccttc
121 ggacccatag atctcaggct cggctccccg cccgccgcag cccactgttg acccggcccg
181 tactgcggcc ccgtggccac c
```

WNK1 (WNK Lysine Deficient Protein Kinase 1/ Serine/threonine-protein kinase WNK1)(4 transcript variants)

Variant 2 ΔG = -531.00 kcal/mol

```
  1 agactcccgg cgccatttag cgcggagagt ttcccgggtg gacgcggctc ctctctcggc
 61 cactccgcac ccccatcttc ggtgacagaa ggcgcctggt gggggtggct gctcttttct
121 ctccctgttc cccctcaccc agtcctctag gtctcctctc ctcttgcctc agagaagcag
181 cggagctcgg gccccgcggt gagcggccct cccctccccg ccgttccctc ctccgtcagc
241 ccccggcacc ggcccgggag gagacgggtt tgccaggcct ggggcgggcg gggaggcctc
301 ggggaagggg gggcccgctc ctcaggcgcc gaggctccga ggctccggcc cttcgcctct
361 gggcgatggg cgacctgtga ggccggtccc catcgctggg ggcgcgtgtg ggaggaggcg
421 gccgcccgag tgaccgggag ccgggccgcg gccttccctc gcccgcctcg gcccctccca
481 ctcctctgcc ccggggccgc caccgcccgg gcgtcggacc tggtcccgtg ctcgcggtgc
541 cgccgccctc tgggcctagc ccgcccagct cggcgagcgg cggcagtggg agccgcgtcc
601 gccgcatccg cctcgactcg gtgccggccc ctggccctcc cctcatgact gcggcgcctc
661 tgctgccacc gcccgcccgg ccgccgctcg ccgcaggatg gatgcggacc gtgcggcgct
721 aacccccgtg gctcagctcc cgaatcgccc gccttcgagc cctcctcgtg agccgcagca
781 gcctcggtgc cagcccccgc cgcagctggg cccagcggtc cgcctgtccc tcgttgcggc
841 ttgtcggtgc tgagtgaggc gtcgtccggg tcggcgcgaa cccgcccggc cgcggttccc
901 tgcagacctc tgcgcgggcg gctcggccct tcacgccctt ttcgttcacg aatccgagcc
961 cgctcgcctc tctccagcga accgacc
```

Variant 1 ΔG = -531.00 kcal/mol

```
  1 agactcccgg cgccatttag cgcggagagt ttcccgggtg gacgcggctc ctctctcggc
 61 cactccgcac ccccatcttc ggtgacagaa ggcgcctggt gggggtggct gctcttttct
121 ctccctgttc cccctcaccc agtcctctag gtctcctctc ctcttgcctc agagaagcag
181 cggagctcgg gccccgcggt gagcggccct cccctccccg ccgttccctc ctccgtcagc
241 ccccggcacc ggcccgggag gagacgggtt tgccaggcct ggggcgggcg gggaggcctc
301 ggggaagggg gggcccgctc ctcaggcgcc gaggctccga ggctccggcc cttcgcctct
361 gggcgatggg cgacctgtga ggccggtccc catcgctggg ggcgcgtgtg ggaggaggcg
421 gccgcccgag tgaccgggag ccgggccgcg gccttccctc gcccgcctcg gcccctccca
481 ctcctctgcc ccggggccgc caccgcccgg gcgtcggacc tggtcccgtg ctcgcggtgc
541 cgccgccctc tgggcctagc ccgcccagct cggcgagcgg cggcagtggg agccgcgtcc
601 gccgcatccg cctcgactcg gtgccggccc ctggccctcc cctcatgact gcggcgcctc
661 tgctgccacc gcccgcccgg ccgccgctcg ccgcaggatg gatgcggacc gtgcggcgct
721 aacccccgtg gctcagctcc cgaatcgccc gccttcgagc cctcctcgtg agccgcagca
781 gcctcggtgc cagcccccgc cgcagctggg cccagcggtc cgcctgtccc tcgttgcggc
841 ttgtcggtgc tgagtgaggc gtcgtccggg tcggcgcgaa cccgcccggc cgcggttccc
901 tgcagacctc tgcgcgggcg gctcggccct tcacgccctt ttcgttcacg aatccgagcc
961 cgctcgcctc tctccagcga accgacc
```

TOM1 (Target Of Myb1 Membrane Trafficking Protein)(7 transcript variants)

Variant 2 ΔG = -61.90 kcal/mol

```
  1 ttcgcggtgc caccgccccg cccacgcctc ctcgccggcc tccgagtgcg tcacgtgacg
 61 ggtcggtggc gctggcggtt gctgtcagct gattcccggg gttggtggca gcggcggtag
121 cagca
```

Variant 1 ΔG = -6.80 kcal/mol

```
  1 gattcccggg gttggtggca gcggcggtag cagca
```

ZNF282 (Zinc Finger Protein 282)(2 transcript variants)

Variant 1 ΔG = -9.20 kcal/mol

```
  1 cttcttctcc ctggccgacc cgagcgggga acagcactcc cagg
```

LRPPRC (Leucine Rich Pentatricopeptide Repeat Containing)(1 transcript variant)

ΔG = -15.00 kcal/mol

```
  1 cttctggcgg agcgtgcttc ccgctgcggg gacgttcgag ca
```

DYNLT1 (Dynein Light Chain Tctex-Type 1)(3 transcript variants)

Variant 2 ΔG = -7.40 kcal/mol

```
  1 actcagggag ccggaggga cgcgccggag gaaag
```

Variant 1 ΔG = -7.40 kcal/mol

```
  1 actcagggag ccggaggga cgcgccggag gaaag
```

S100A11 (S100 Calcium Binding Protein A11)(1 transcript variant)

ΔG = -23.00 kcal/mol

```
  1 gaggagaggc tccagacccg cacgccgcgc gcacagagct ctcagcgccg ctcccagcca
 61 cagcctcccg cgcctcgctc agctccaac
```

ISG15 (ISG15 Ubiquitin Like Modifier)(1 transcript variant)

ΔG = -27.40 kcal/mol

```
  1 ggcggctgag aggcagcgaa ctcatctttg ccagtacagg agcttgtgcc gtggcccaca
 61 gcccacagcc cacagcc
```

TOMM20 (Translocase Of Outer Mitochondrial Membrane 20)(1 transcript variant)

ΔG = -43.60 kcal/mol

```
  1 ctttctgtgt tcctggcccg cggccgtcgg gtgtgagctg cgccgaccgc tctgagggtt
 61 cgtggcccac cgctccttcg cggtccctgc cgccaccgtc cacgctcagc gttgtagaga
121 ag
```

TRIAP1 (TP53 Regulated Inhibitor Of Apoptosis 1/ P53-Inducible Cell-Survival Factor)(1 transcript variant)

ΔG = 3.00 kcal/mol,

```
        1 accactgtcg cc
```

OGDH (Oxoglutarate Dehydrogenase)(4 transcript variants)

Variant 1 ΔG = -12.20 kcal/mol

```
        1 attcgggtgg agctgagccg gagacaggca gttgtgaaaa acttcaggac aaaa
```

MECR (Mitochondrial Trans-2-Enoyl-CoA Reductase)(13 transcript variants)

Variant 1 ΔG = -6.60 kcal/mol

```
        1 gtcaggctct gtgttggttg gagcgagc
```

COMMD9 (COMM Domain Containing 9)(4 transcript variants)

Variant 4 ΔG = -17.60 kcal/mol

```
        1 gacttcggca agatggctgc cctgacagcg gagcattttg cagcactcca gagcctgctc
       61 aaga
```

Variant 1 ΔG = 2.00 kcal/mol

```
        1 gacttcggca ag
```

SYNGR2 (Synaptogyrin 2)(3 transcript variants)

Variant 1 ΔG = -15.70 kcal/mol

```
        1 gcgttccgcg gcggcggcag cggcggcgac ggcgac
```

PGK1 (Phosphoglycerate Kinase 1/ Cell Migration-Inducing Gene 10 Protein)(1 transcript variant)

ΔG = -21.50 kcal/mol

```
        1 gttccgcatt ctgcaagcct ccggagcgca cgtcggcagt cggctccctc gttgaccgaa
       61 tcaccgacct ctctccccag ctgtatttcc aaa
```

GYS1 (Glycogen Synthase 1)(3 transcript variants)

Variant 1 ΔG = -79.70 kcal/mol

```
        1 agtgacgctg cggcctcctt ctgcctaggt cccaacgctt cggggcaggg gtgcggtctt
       61 gcaataggaa gccgagcgtc ttgcaagctt cccgtcgggc accagctact cggccccgca
      121 ccctacctgg tgcattccct agacacctcc ggggtcccta cctggagatc cccggagccc
      181 cccttcctgc gccagcc
```

PEF1 (Peflin/ Penta-EF-Hand Domain Containing 1)(3 transcript variants)

Variant 1 ΔG = 2.80 kcal/mol

```
        1 gtcagaatca cc
```

DUSP23 (Dual specificity protein phosphatase 23)(3 transcript variants)

Variant 1 ΔG = -73.00 kcal/mol

```
  1 agaccacgtg gcccgggagg cgccgaggcc aggtaggtgg tgagttactt ggctcggagc
 61 gggcgagggg acgcgtgggc ggagcggggc tggccagcct cggccccccat gacccgctgt
121 cctgtgccct ttcccagcg
```

CHST3 (Carbohydrate Sulfotransferase 3)(1 transcript variant)

ΔG = -215.90 kcal/mol

```
  1 gctagccggc tcgggcctga gcggggaggg cgccaggcag gacctctcgc aggctcgctg
 61 cgcaggacgg cgcccgcctg gcgccgcttc ccttccagcg tgccgaccgg ccccgcagcg
121 cctccatccc tccggcccgc cccggagaag acgcacagct cgggccgcgc gggcgccggg
181 gccgcggaac cgcttctgcc gggatcttcc aggaggaaag cgaagttgcg agcggatgct
241 gcccgcgccg gaccccagcc gcggagggtc ggggccgccg gtggagtctc ggcggccggg
301 gacaagggtg tcccccacct gaagacggca agctgggtcc tgagtgatgc ccctcagctg
361 agtgtccaag gctggcccga ggagccccca cggccccacc tttcccc
```

THY1 (Thy-1 Membrane Glycoprotein)(4 transcript variants)

Variant 1 ΔG = -29.10 kcal/mol

```
  1 agcaaccgga ggcggcggcg cgtctggagg aggctgcagc agcggaagac cccagtccag
 61 atccaggact gagatcccag aacc
```

SCARB1 (Scavenger receptor class B member 1)(20 transcript variants)

Variant 1 ΔG = -66.40 kcal/mol

```
  1 cctgcgtgcg ctgccgtccc ggatccaccg tgcctctgcg gcctgcgtgc ccggagtccc
 61 cgcctgtgtc gtctctgtcg ccgtccccgt ctcctgccag gcgcggagcc ctgcgagccg
121 cgggtgggcc ccaggcgcgc agac
```

INF2 (Inverted Formin, FH2 And WH2 Domain Containing)(3 transcript variants)

Variant 1 ΔG = -62.50 kcal/mol

```
  1 gccaggagcc accgtccgag ccttgcggag cgcggcagtg ggcgccggct gcccgcagcc
 61 cctgacccgg ccccggacgg agcgccggcc gcaccaccgc cctctggccg ttgcctcacc
121 ggctcggcaa g
```

LEPREL4 (P3H4 = Prolyl 3-Hydroxylase Family Member 4 (Non-Enzymatic))(1 transcript variant)

ΔG = -13.30 kcal/mol

```
  1 agctcagctc cggagagccg gcggcgcggc gggc
```

PTRF (Polymerase I And Transcript Release Factor /CAVIN1 = Caveolae Associated Protein 1)(1 transcript variant)

ΔG = -48.70 kcal/mol

```
  1 agttctggcc gctgtcccgg tgcgcacgga cgtggctcga gtttcctctg ctctccgctc
 61 tcgcccgcta gctctcctcc cttccgctcc tgcttctctc cgggtctccc gctccagctc
121 cagccccacc cggccggtcc cgcacggctc cgggtagcc
```

CHMP6 (Charged Multivesicular Body Protein 6)(1 transcript variant)

ΔG = -40.00 kcal/mol

```
  1 aggacccgag ctacggtggc cgcggggcgg cggtggcgat tggacttggt gggtcccggg
 61 ccaggggcgg gcgccgcc
```

FASN (Fatty acid synthase)(1 transcript variant)

ΔG = -34.40 kcal/mol

```
  1 gagccagaga gacggcagcg gccccggcct ccctctccgc cgcgcttcag cctcccgctc
 61 cgcccgcgctc cagcctcgct ctccgccgcc cgcaccgccg cccgcgccct caccagagca
121 gcc
```

CCBE1 (Collagen and calcium-binding EGF domain-containing protein 1)(1 transcript variant)

ΔG = -33.70 kcal/mol

```
  1 gaaggcggcg gcggccgcgg aggagcagga cgcttggtcc ggacggagct cggcgctggg
 61 aagaagccgg gagcttccct g
```

TMEM259 (Transmembrane Protein 259/ Membralin)(2 transcript variants)

Variant 2 ΔG = -59.70 kcal/mol

```
  1 gagaaagcgg aagatggcgg cggcggccgg gaggccgtga ggagagcggc ggctgcgagg
 61 gcggccgatg gcggccggga ggcgccctcg gacacttgcg ggtcgttagg gcgcgacgct
121 gggaggc
```

Variant 1 ΔG = -59.70 kcal/mol

```
  1 gagaaagcgg aagatggcgg cggcggccgg gaggccgtga ggagagcggc ggctgcgagg
 61 gcggccgatg gcggccggga ggcgccctcg gacacttgcg ggtcgttagg gcgcgacgct
121 gggaggc
```

OAZ1 (Ornithine decarboxylase antizyme 1)(2 transcript variants)

Variant 2 ΔG = -35.80 kcal/mol

```
  1 agcatctata aaggcgggcg gcggcagagg cgccattttg cgaacggcga gcagcggcgg
 61 cggcgcggag agacgcagcg gaggttttcc tggtttcgga ccccagcggc cgg
```

Variant 1 ΔG = -35.80 kcal/mol

```
  1 agcatctata aaggcgggcg gcggcagagg cgccattttg cgaacggcga gcagcggcgg
 61 cggcgcggag agacgcagcg gaggttttcc tggtttcgga ccccagcggc cgg
```

MFSD12 (Major facilitator superfamily domain-containing protein 12)(4 transcript variants, variant 1 not available on NCBI)

Variant 3 ΔG = -93.60 kcal/mol

```
  1 gcagagtcga gcgtggggcg gacgcggcgg acgtgggtga gggcgcggcc gtaagagagc
 61 gggacgcggg gtgcccggcg cgtggtgggg gtccccggcg cctgccccca cggcacccaa
121 gaaggcctgg ccagggtacc ctccgcggag cccgggggtg gggggcgcgg gcccggcgcc
181 gcg
```

PLIN3 (Perilipin-3)(3 transcript variants)

Variant 3 ΔG = -26.70 kcal/mol

```
  1 ggtttccaag ctggttttga agtcgcggca gctgttcctg ggacgtccgg ttgaccgcgc
 61 gtctgctgca gagacc
```

Variant 1 ΔG = -26.70 kcal/mol

```
  1 ggtttccaag ctggttttga agtcgcggca gctgttcctg ggacgtccgg ttgaccgcgc
 61 gtctgctgca gagacc
```

WDR83OS (WD Repeat Domain 83 Opposite Strand/ Protein Asterix)(1 transcript variant)

ΔG = 2.80 kcal/mol

```
  1 cttcgactcg ct
```

TMEM161A (Transmembrane Protein 161A)(2 transcript variants)

Variant 1 ΔG = -15.80 kcal/mol

```
  1 gcccggagcc ctaggccggt gggtgagtgc accgcgttct cgcacgcgtc
```

PEPD (Peptidase D/ Xaa-Pro Dipeptidase)(3 transcript variants)

Variant 2 ΔG = -7.50 kcal/mol

```
  1 gcacttcacg tgacgccggt gccgggcgaa c
```

Variant 1 ΔG = -7.50 kcal/mol

```
  1 gcacttcacg tgacgccggt gccgggcgaa c
```

GPI (Glucose-6-Phosphate Variantmerase)(7 transcript variants)

Variant 3 ΔG = -44.80 kcal/mol

```
  1 aatagccctt accaccagca gacacacatc atctgttgta cttgcttatt tggcacatat
 61 gtatccacag cgcctagaac actgcctgta acgtggaagg tgttcgatct atagagtttt
121 gtcgaatgaa tgaatgaagc cgactagtgc acagggagtg cagcggcgcg
```

Variant 1   ΔG = -44.80 kcal/mol

```
  1 aatagccctt accaccagca gacacacatc atctgttgta cttgcttatt tggcacatat
 61 gtatccacag cgcctagaac actgcctgta acgtggaagg tgttcgatct atagagtttt
121 gtcgaatgaa tgaatgaagc cgactagtgc acagggagtg cagcggcgcg
```

GPC1 (Glypican Proteoglycan 1)(1 transcript variant)

ΔG = -140.70 kcal/mol

```
  1 gccgagccgg gactgcgcta gcccgccgcg ctctgggctg cccgagcgag cgttcggacc
 61 tcgcaccccg cgcgccccgc gccgccgccg ccgccggctt ttgttgtctc cgcctcctcg
121 gccgccgccg cctctggacc gcgagccgcg cgcgccggga ccttggctct gcccttcgcg
181 ggcgggaact gcgcaggacc cggccaggat ccgagagagg cgcgggcggg tggccggggg
241 cgccgccggc cccgcc
```

ITPA (Inosine triphosphate pyrophosphatase)(10 transcript variants)

Variant 3   ΔG = -17.40 kcal/mol

```
  1 gtcactggac gccaaggagt tttcggtggc tcagctgggt aaccggggat cacc
```

Variant 1   ΔG = -17.40 kcal/mol

```
  1 gtcactggac gccaaggagt tttcggtggc tcagctgggt aaccggggat cacc
```

UQCR10 (Ubiquinol-Cytochrome C Reductase, Complex III Subunit X/ Cytochrome b-c1 complex subunit 9)(2 transcript variants)

Variant 1   ΔG = -6.70 kcal/mol

```
  1 gcggtggcgc gagttggact gtgaagaaac
```

RNF7 (Ring Finger Protein 7/ RING-box protein 2)(6 transcript variants)

Variant 1   ΔG = -11.50 kcal/mol

```
  1 cttccccaag ccaacgtctc cgccgtcggc tccgcggcgc cgcc
```

ACOX3 (Acyl-CoA Oxidase 3, Pristanoyl/ Peroxvariantmal acyl-coenzyme A oxidase 3)(2 transcript variants)

Variant 1   ΔG = -25.10 kcal/mol

```
  1 aaatgagaat ggcgccgctg gagatccttt cctgcttttg gtttccctgg caggggttga
 61 actgtggagt gtgtgggctc ttatcacgcg
```

DDX41 (DEAD-Box Helicase 41/ Probable ATP-dependent RNA helicase DDX41)(3 transcript variants)

Variant 2   ΔG = -288.30 kcal/mol

1 atgcgtgcag caaagaatgg aggagtcgga acccgaacgg aagcgggctc gcaccgacga
61 ggtgcctgcc ggaggaagcc gctccgaggc ggaagatgag gacgacgagg actacgtgcc
121 ctatgtgccg ttacggcagc gccggcagct actggtgagg ggcacgcgtc cggggaggga
181 tggggccgtg accgcggggt cgcagggggc gggcgtggcc tcggcctccg tggggaggag
241 ggcagtctga ggagtgagct tcagtacacc agggcgcggc ccccagacaa acgagggacc
301 gacggcttga tctgaggggt agggcagctt tgcgcctcgg gcacaggatc ctcgccccaa
361 agccaggatc ctgacgctga ccttgtggac tccatgccca tcctatctgt ggatcccggg
421 tggaacctca gctccagaag ctgctgcagc gaagacgcaa gggagctgcg gaggaagagc
481 agcaggacag cggtagtgaa ccccggggag atgaggacga catcccgcta ggccctcagt
541 ccaacgtcag cctcctggat cagcaccagc accttaaaga gaaggctgaa gcgcgcaaag
601 agtctgccaa ggagaagcag ctgaaggaag aagagaagat cctggagagt gttgccgagg
661 gccgagcatt g

Variant 1 ΔG = -2.70 kcal/mol

1 atgcgtgcag caaaga

PMPCA (Peptidase, Mitochondrial Processing Alpha Subunit)(3 transcript variants)

Variant 3 ΔG = -138.20 kcal/mol

1 aaagcgcgtg cctgaacgcc ttgggccgtc ggcgaggggg aggggaagcc gtgggcggaa
61 gcggaagtga cgactgaagc ggggcggaga cgcaagatgg cggctgtggt gctggcggcg
121 acgcggttgc tgcggggctc gggttcttgg ggctgttcgc ggctgaggtt tggacctcct
181 gcgtacagac ggtttagtag tggtggtgcc tatcccaaca tccccctctc ttctccctta
241 cctggagtac ccaagcctgt ttttgctaca gttgatggac aggaaaagtt tgaaaccaaa
301 gtaaccacat tggataatgg gcttcgcgtg gcatctcaga ataagtttgg acagttttgt
361 acagtaggaa ttcttatcaa ttcaggatcg agat

Variant 1 ΔG = /

1 ggagacgcaa g

GUK1 (Guanylate Kinase 1)(5 transcript variants)

Variant 5 ΔG = -5.90 kcal/mol

1 agtgggcgga agaggtggcc ccgg

Variant 1 ΔG = -24.70 kcal/mol

1 agtctcgcgc gcgggcggag gtgggccggt gcggcgctgt cacgtaggtt cagtgggcgg
61 aagaggtggc cccgg

ALDH9A1 (Aldehyde Dehydrogenase 9 Family Member A1/ 4-trimethylaminobutyraldehyde dehydrogenase)(2 transcript variants)

Variant 2 ΔG = -151.70 kcal/mol

1 actcatgttt ctccgagcag gcctggccgc gctctccccg cttcttcgca gtcttcggcc
61 ctctcctgtc gccgccatga gcactggcac cttcgtcgtg tcgcagccgc tcaattaccg
121 cggcggggcc cgcgtggagc cggcggacgc ctccggtacc gagaaagctt tcgagccagc
181 aaccggtaac tgcaacgagc cggggaggct ggggcgccct ggggccggat gcgcggattt
241 cccggccagc ccccgtttcc tgtgttctgc agcgttgact tgagcacaag acagtgacag
301 tggagagtct aaggccgagt gatagctact ttcacatgtt caggagaaaa ggaagtaaat

```
361 ttggctgttc aaaatgcaaa ggctgctttt aaaatatgga gtcaaaaatc tggc
```

Variant 1 ΔG = -30.90 kcal/mol

```
  1 ctgcccctaa aatctcctag aaccgatccc gcggccccgc ccctcccgcg gccccgcccc
 61 tcccgcggcc cgtcagcctc tgccgcggag ctgcgtccgc cactc
```

DHCR7 (7-Dehydrocholesterol Reductase)(2 transcript variants)

Variant 2 ΔG = -85.50 kcal/mol

```
  1 gcgggtgact ccgacccctg gctagagggt aggcggcgtg gagcagcgcg cgcaagcgag
 61 gccaggggaa ggtgggcgca ggactttagc cggttgagaa ggatcaagca ggcatttgga
121 gcacaggtgt ctagaaactt ttaaggggcc ggttcaagaa ggaaaagttc ccttctgctg
181 tgaaactatt tggcaagagg ctggagggcc ca
```

Variant 1 ΔG = -119.80 kcal/mol

```
  1 aatcgctgac atcatccggg ggcgggcgcc cctgccctgc gggtgactcc gacccctggc
 61 tagagggtag gcggcgtgga gcagcgcgcg caagcgaggc caggggaagg tgggcgcagg
121 tgaggggccg aggtgtgcgc aggactttag ccggttgaga aggatcaagc aggcatttgg
181 agcacaggtg tctagaaact tttaaggggc cggttcaaga aggaaaagtt cccttctgct
241 gtgaaactat ttggcaagag gctggagggc cca
```

ATP6V0D1 (ATPase H+ Transporting V0 Subunit D1/ V-type proton ATPase subunit d 1)(1 transcript variant)

ΔG = -32.30 kcal/mol

```
  1 agctgacctg gggagtcgcg attcgtgccg gccggtcctg gttctccggt cccgccgctc
 61 ccgcagcagc c
```

GTF3C2 (General Transcription Factor IIIC Subunit 2)(3 transcript variants)

Variant 3 ΔG = -155.00 kcal/mol

```
  1 ggtaacgcgg aagcctgtgt ccgggcgtag gcgctgcata cgtagcgtcc ctcgaactcg
 61 gcgaacctac agtagtcctt gttagaaaaa ccagggctcc tctcccgcac aggctagggg
121 agaaatctac cttcacgccc aggactgaag aagggcaggt gatggcccct agagtggttg
181 tgggtcaatc tctttcagct gtgtcccagc ctcttcctcg aggaggctct ttaaccccga
241 gggttgtgcc ccgcgcgctg caacgccatt ggcgtaacca agggccagtt gtgtttccct
301 gtctagtcct tttgtcccgt ggcaagttgg ctgcctgttg gggaagtcag aggtggcgac
361 ttcaactccg tcggaggcaa ctcactttgt tggaggg
```

Variant 1 ΔG = -143.90 kcal/mol

```
  1 cccttttga cactgcaact cccatgaggc cctgcagcgt ccctggcgcc gctgaagacg
 61 ccctggaact cggctggctt cggagggctc ggtgactctg gccgcgctgc attatggaat
121 acagagtcta agagaaaact agcccggcac cgactccgga ccaagcacac gtgtttacct
181 tgtgcactgg ggtgggggcg tcagtgaaga gcagctaaca agatttcgtc ataagaccgc
241 gaccagacag gcggcgccat cttcgaactt agacttccgg aaggactttg gcgaggcttg
301 agtgtgcgtc agaatcccct gaagagcttg ttaaaacaca aggtgctggc cccatccaga
361 attgctgatt cggggcagcc attttggggg gtgctg
```

MAEA (Macrophage Erythroblast Attacher/ E3 ubiquitin-protein transferase MAEA)(6 transcript variants)

Variant 6 ΔG = -105.10 kcal/mol

```
  1 actgggagca aagggttcct gtctgcagca caccagctgc ccggagctct gccctgtgcc
 61 tgagcagcgt cagcaccgcg gtgaggcgga cggcccggca ggggttggaa agtccagaaa
121 ggccccgttc ccgcgtagaa cttgaatgca gctaagggac gtccagagag gcctttcctt
181 ttctggtggc ggtctggaat gtgctggtgc cctacgagac gctgaacaaa cgctttcgcg
241 ccgctcagaa gaacattgac cgggagacca gccacgtcac c
```

Variant 1 ΔG = -3.70 kcal/mol

```
  1 gctaatgttt tggccgcttc aag
```

VCP (Valosin Containing Protein/ Transitional Endoplasmic Reticulum ATPase)(3 transcript variants)

Variant 1 ΔG = -120.70 kcal/mol

```
  1 agtctcagcg aagcgtctgc gaccgtcgtt tgagtcgtcg ctgccgctgc cgctgccact
 61 gccactgcca cctcgcggat caggagccag cgttgttcgc ccgacgcctc gctgccggtg
121 ggaggaagcg agagggaagc cgcttgcggg tttgtcgccg ctgctcgccc accgcctgga
181 agagccgagc cccggcccag tcggtcgctt gccaccgctc gtagccgtta cccgcgggcc
241 gccacagccg ccggccggga gaggcgcgcg cc
```

ALDOA (Aldolase, Fructose-Bisphosphate A/ Fructose-bisphosphate aldolase A)(6 Transcript variants; variant 1 not available on NCBI)

Variant 6 ΔG = -61.90 kcal/mol (longest)

```
  1 gcccgcctcc tgcgccgccc cttccgaggc taaatcggct gcgttcctct cggaacgcgc
 61 cgcagaaggg gtcctggtga cgagtcccgc gttctctcct tgaatccact cgccagcccg
121 ccgccctctg ccgccgcacc ctgcacaccc gcccctctcc tgtgccaggc aaggtgaccc
181 c
```

Boxplots

RPF-changed (padj<0.05)_mRNA-unchanged (resLA_padj<0.1) [kcal/mol]

pos.(rep.24): -109.4, -45.6, -106.1, -176.7, -24.1, -57.8, -111.2, -78.0, -192.6, -52.80, -117.20, -35.50, -40.30, 2.20, -47.90, -76.30, -23.60, -60.90, -77.80, -52.70, -206.00, -166.40, -16.10, -53.60

neg.(act.10): -32.7, -77.3, -7.6, -34.80, -13.40, -56.60, -21.00, -47.70, -19.20, -2.90

mRNA-changed (padj<0.05)_RPF-unchanged (resLA_padj<0.1) [kcal/mol]

pos.(act.48): -29.1, -61.9, -59.7, -143.9, -66.4, -33.7, -30.9, -140.7, -7.5, 2.8, -62.5, 2.8, -120.7, -6.7, -44.8, -32.3, -11.5, -3.7, -26.7, -2.7, 0.0, -15.8, -13.3, -24.7, -40.0, -34.4, -17.4, -93.6, -48.7, -25.1, -35.8, -215.9, -73, -119.8, -6.80, -15.00, -7.40, -9.20, -23.00, -27.40, -43.60, 3.00, -12.20, -6.60, 2.00, -15.70, -21.50, -79.70

neg.(rep.38): -514.3, -96.5, -67.1, -125.4, -315.2, -156.8, -155.8, -70.4, -88.6, -66.8, -147.1, -110.1, -195.7, -112.9, -117.4, -531.0, -76.0, -148.0, -124.2, -406.30, -117.00, -11.40, -333.50, -106.10, -62.80, -57.20, -20.70, -37.90, -44.90, -95.70, -96.60, -120.50, -18.50, -46.60, -235.40, -244.30, -118.50, -50.20

all repressed by Pdcd4

-109.4, -45.6, -106.1, -176.7, -24.1, -57.8, -111.2, -78.0, -192.6, -52.80, -117.20, -35.50, -40.30, 2.20, -47.90, -76.30, -23.60, -60.90, -77.80, -52.70, -206.00, -166.40, -16.10, -53.60, -514.3, -96.5, -67.1, -125.4, -315.2, -156.8, -155.8, -70.4, -88.6, -66.8, -147.1, -110.1, -195.7, -112.9, -117.4, -531.0, -76.0, -148.0, -124.2, -406.30, -117.00, -11.40, -333.50, -106.10, -62.80, -57.20, -20.70, -37.90, -44.90, -95.70, -96.60, -120.50, -18.50, -46.60, -235.40, -244.30, -118.50, -50.20

all activated by Pdcd4

-32.7, -77.3, -7.6, -34.80, -13.40, -56.60, -21.00, -47.70, -19.20, -2.90, -29.1, -61.9, -59.7, -143.9, -66.4, -33.7, -30.9, -140.7, -7.5, 2.8, -62.5, 2.8, -120.7, -6.7, -44.8, -32.3, -11.5, -3.7, -26.7, -2.7, 0.0, -15.8, -13.3, -24.7, -40.0, -34.4, -17.4, -93.6, -48.7, -25.1, -35.8, -215.9, -73, -119.8, -6.80, -15.00, -7.40, -9.20, -23.00, -27.40, -43.60, 3.00, -12.20, -6.60, 2.00, -15.70, -21.50, -79.70

Original western blots

PDCD4 controls the G1/S-phase transition in a telomerase-immortalized epithelial cell line and affects the expression level and translation of multiple mRNAs

Astrid Haas, Benedikt S. Nilges, Sebastian A. Leidel, Karl-Heinz Klempnauer

Original western blots for Figure 1a

short exp.

top panel — anti Pdcd4

bottom panel — anti β-actin

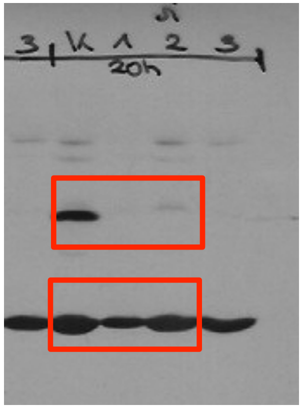

lanes 1 2 3

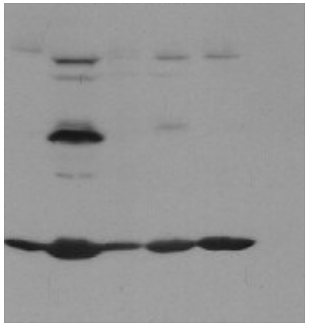

middle exp.

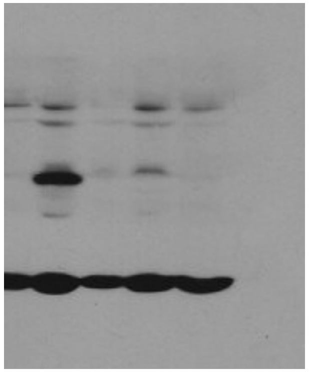

lobger exp.

Original western blots for Figure 2a

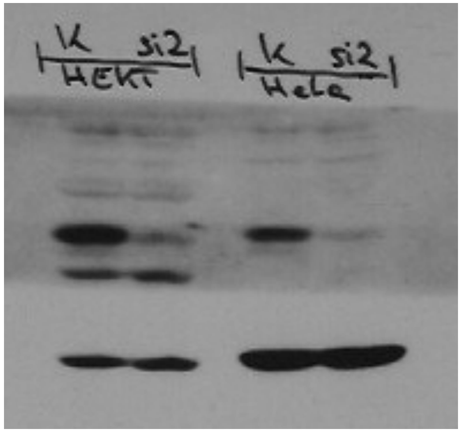

longer exp.

top panel

anti Pdcd4

bottom panel

anti β-actin

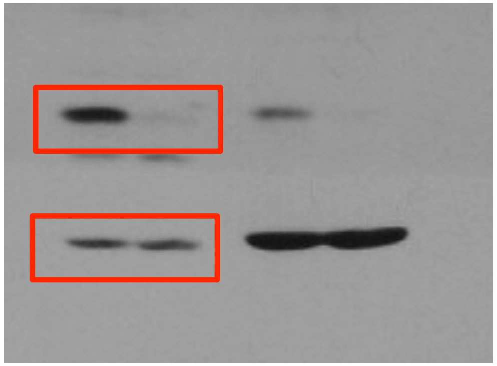

lanes 1 2

middle exp.

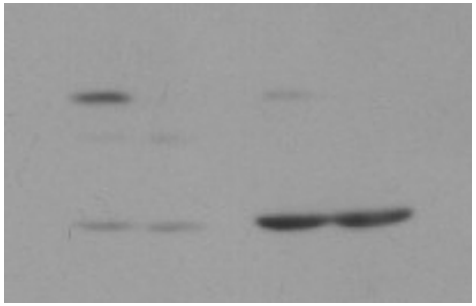

shorter exp.

Original western blots for Figure 3a

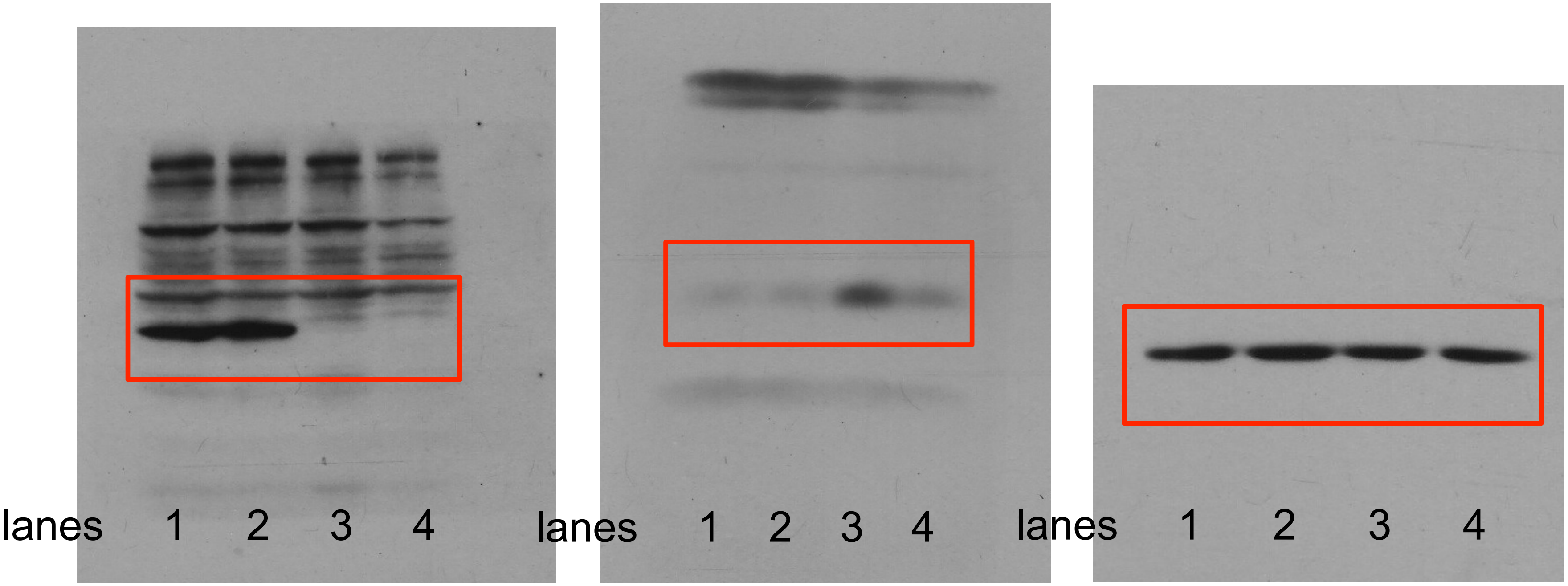

top panel
anti Pdcd4

middle panel
anti p21

bottom panel
anti β-actin

Original western blots for Figure 3c

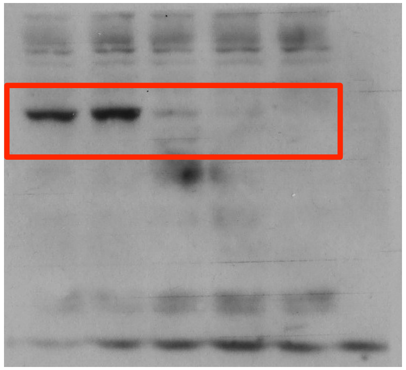

top panel
anti Pdcd4

lanes 1 2 3 4 5

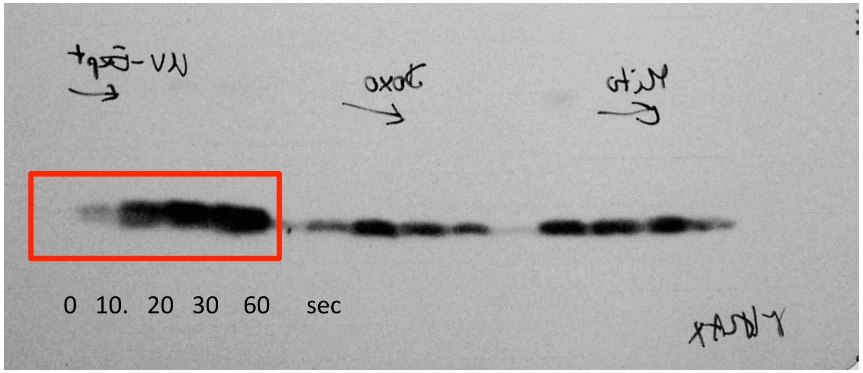

middle panel
γ-H2AX

lanes 1 2 3 4 5

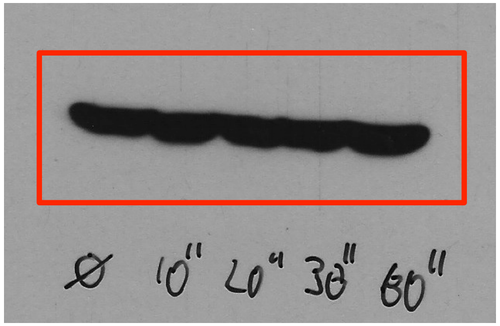

bottom panel
β-actin

lanes 1 2 3 4 5

Original western blots for Figure 3d

op 4D
Pdcd4

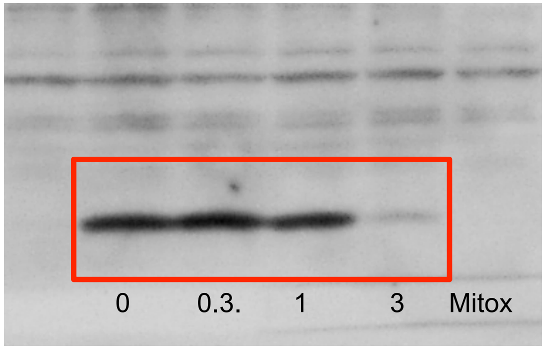

top panel
anti Pdcd4

lanes 1 2 3 4

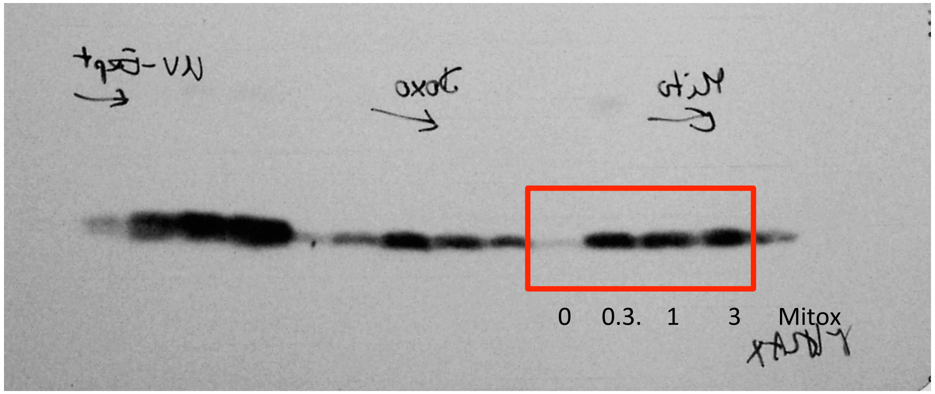

middle panel
γ-H2AX

lanes 1 2 3 4

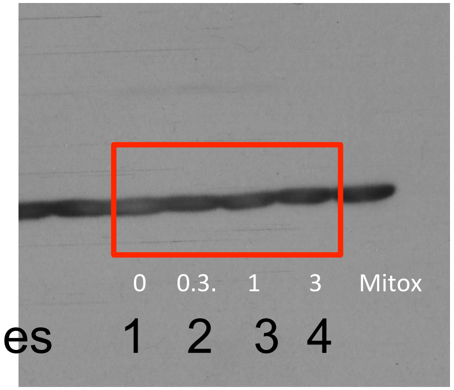

bottom panel
anti β-actin

Original western blots for supplementary Figure 1b

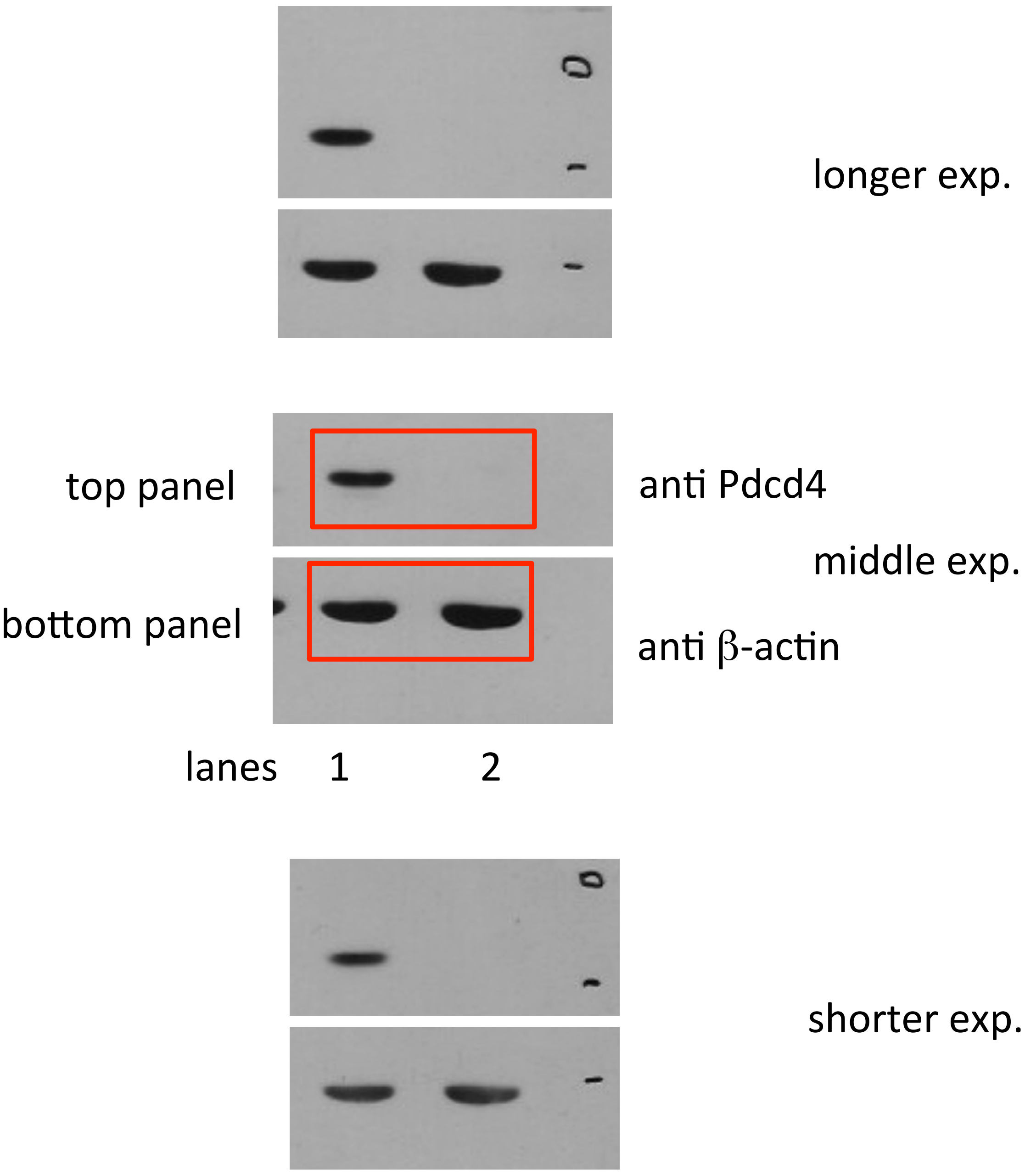

Supplement: Supplementary file 1 — Supplementary data. [file 41598_2020_59678_MOESM1_ESM.pdf]
